# Symbiotic N_2_ Fixation, Leaf Photosynthesis, and Abiotic Stress Tolerance of Native Rhizobia Isolated from Soybean Nodules at Da, Upper West Region, Ghana

**DOI:** 10.3390/microorganisms13040876

**Published:** 2025-04-11

**Authors:** Mmatladi Tesia Mataboge, Mustapha Mohammed, Felix Dapare Dakora

**Affiliations:** 1 Department of Crop Sciences, Tshwane University of Technology, Private Bag X680, Pretoria 0001, South Africa; daisymnguni@gmail.com; 2 Department of Crop Science, University for Development Studies, Tamale P.O. Box TL 1882, Ghana; mmutapha@uds.edu.gh; 3 Department of Chemistry, Tshwane University of Technology, Private Bag X680, Pretoria 0001, South Africa

**Keywords:** root nodulation, rhizobia, ^15^N/^14^N isotopes, ^13^C/^12^C, symbiotic effectiveness

## Abstract

The soybean is an important source of protein and is gaining popularity in Ghana due to a rising demand for its use in the poultry industry. However, the grain yield of soybeans is relatively low in the Upper West Region due to infertile soil and climate change. This study evaluated root nodulation and symbiotic effectiveness in 31 rhizobial isolates obtained from the nodules of soybeans planted at Da in the Upper West Region, Ghana, as well as measured photosynthetic activity of the soybean plants grown under glasshouse conditions. This study further assessed the tolerance of the rhizobial isolates to different levels of temperature, drought, salinity, and pH in the laboratory and also measured the ability of the isolates to produce indole-3-acetic acid. An infrared gas analyser and the ^15^N and ^13^C natural abundance techniques were used to assess the photosynthetic activity, N_2_ fixation, and water-use efficiency, respectively. The results showed that the test isolates that induced greater photosynthetic rates from higher stomatal conductance also stimulated increased water loss via leaf transpiration in soybean plants. Isolates TUTGMGH9 and TUTGMGH19 elicited much higher shoot δ^13^C in the soybean host plant and induced higher shoot biomass, C accumulation, percent relative symbiotic effectiveness, and N_2_ fixation relative to *Bradyrhizobium* strain WB74 and 5 mM of nitrate, which were used as positive controls. Although isolate TUTGMGH9 did not grow at 40 °C, it showed growth at 5% of PEG-6000, NaCl, and a low pH while also producing moderate IAA. However, for better utilisation of these rhizobial isolates as bioinoculants, their growth performance needs to be assessed under field conditions to ascertain their competitiveness and symbiotic efficacy.

## 1. Introduction

The soybean (*Glycine max* L. Merr.) is a nutritionally important grain legume in the world because of its high protein (40%), oil content (20%), and low cholesterol, as well as its high dietary fibre [1]. In Ghana, soybeans have gained popularity partly due to the increase in demand from the poultry and oil industries [2,3]. Although soybean production in Ghana is relatively low [4], the northern parts of the country account for about 96% of the crop’s output, making its cultivation a major source of livelihood in the region [2].

Over the past decade, the soybean–rhizobia symbiosis has become important due to the role of symbiotic N_2_ fixation as a component in sustainable and environmentally friendly green agriculture [5]. However, the optimisation of N_2_ fixation in legumes such as soybeans requires the use of effective microsymbionts that are adapted to prevailing edaphoclimatic conditions [6,7]. Because ineffective indigenous soil rhizobia often outcompete introduced strains for nodule occupancy in field-grown legumes, several attempts to use rhizobial inoculants as biofertilisers have often failed [8,9]. In Africa, however, there are many reports of inoculation success with rhizobial application to field-grown legumes. For example, in Mozambique, inoculating promiscuous-nodulating soybeans with a *Bradyrhizobium* strain increased plant growth, N_2_ fixation, and grain yield by 32, 64, and 12%, respectively [10]. In Ethiopia, common bean inoculation with the *Rhizobium phaseoli* strain HB-429 at Galalicha increased plant growth, %Ndfa, amount of N-fixed, and grain yield by 19, 17, 54, and 48%, respectively, over uninoculated control [11]. Similarly, inoculating beans with the *Rhizobium* strain HB-429 at Hawassa increased shoot biomass, nodule number per plant, nodule dry matter, and grain yield per hectare by 9, 40, 54, and 49%, respectively, in 2012, and by 20, 39, 13, and 69%, respectively, in 2013 [12]. In Ghana, applying the *Bradyrhizobium* strain CB756 or the *Bradyrhizobium* strain BR 3267 to Kersting’s groundnut increased shoot growth, N-fixed, and grain yield of the landraces Dowie and Heng MM at Savelugu, and the landraces Heng MM and Sigiri at Gbalahi, compared to the uninoculated control [13]. Furthermore, in Ghana, inoculating cowpea at different locations yielded benefits, as found with applying the *Bradyrhizobium* strain BR3267 to variety Zayura, which increased shoot biomass, N-fixed, and grain yield compared to the uninoculated control, but was decreased with the inoculant strain CB756 at Gbalahi [14]. However, at Savelugu, strain CB756 induced a marked increase in shoot growth, N-fixed, and grain yield of variety Bawutawuta, in contrast to Songotra, which recorded greater shoot biomass, higher amounts of N-fixed, and increased grain yield with the *Bradyrhizobium* strain BR 3267.

Indigenous rhizobia are generally better adapted to local edaphoclimatic conditions than exotic commercial inoculants that often exhibit low effectiveness when transferred from the laboratory to the field due to competition with native rhizobia for the establishment of symbiosis [15,16]. Thus, the competitive ability of introduced rhizobial strains against indigenous soil microbes for nodule occupancy is critical for determining inoculation success in the field [17]. Unfortunately, abiotic stresses have detrimental constraints on symbiotic N_2_ fixation and the yield of soybeans, among other crops. Ghana is in a tropical region with erratic rainfall and high temperatures, both of which contribute to the occurrence of drought and soil acidity [18]. The optimum temperature for rhizobial growth is 28 °C, and an increase in soil temperature above this value can increase evapo-transpiration rates and, consequently, cause a reduction in rhizobial growth, rate of root colonisation, and nodule biomass [16,19]. Salinity is prevalent in most arid and semiarid regions and can limit the transport of solutes from the root zone to shoots due to insufficient soil moisture. Soil salinity can also affect the legume–rhizobia symbiotic interaction by interfering with the infection process and rhizobial survival, as a result of toxicity and osmotic stress [20,21].

With the changing climate that has characterised most parts of the world, the inoculation of legumes with efficient and stress-tolerant rhizobia is a promising strategy for the sustainable production of grain legumes. For example, the inoculation of legumes with salt-tolerant [22,23], heat-resistant [24], cold-tolerant [25], drought-tolerant [26], and acid (low pH) tolerant rhizobia [27] has been shown to increase the yield of legumes. Thus, there are rhizobial strains that possess intrinsic mechanisms for plant growth promotion and abiotic stress tolerance, including the synthesis of indole acetic acid (IAA) and exopolysaccharides [16]. The search for effective and naturally adapted rhizobial strains for inoculant production often entails the characterisation of native rhizobial isolates for their symbiotic effectiveness and adaptability to changing environmental conditions.

Furthermore, the ability of rhizobia to fix N_2_ biologically can significantly enhance the photosynthetic efficiency of nodulated legumes, as nitrogen is a key component in the chlorophyll molecule needed for harvesting light energy during photosynthesis, and the Rubisco enzyme, which is important for reducing CO_2_ to carbohydrates [28]. Grain legumes inoculated with effective rhizobial strains generally exhibit higher photosynthetic rates due to increased chlorophyll and Rubisco biosynthesis. Studies of Bambara groundnut, Kerstings’ groundnut, and cowpea in Ghana, South Africa, and Eswatini have, for example, shown that high N_2_-fixing rhizobia boosted photosynthetic functioning, leading to greater shoot biomass and higher grain yields [28,29,30,31].

The aim of this study was to (i) assess the root nodulation and symbiotic efficiency of soybean rhizobial isolates obtained from Da in the Upper West Region, Ghana, (ii) determine photosynthetic rates of soybean plants as a measure of N_2_-fixing effectiveness of the microsymbionts, (iii) assess the tolerance of rhizobial strains to different levels of temperature, drought, salinity, and pH in the laboratory, and (iv) screen the bacterial isolates for their ability to produce indole-3-acetic acid (IAA) under laboratory conditions.

## 2. Materials and Methods

### 2.1. Study Sites and Nodule Sample Collection

The bacterial isolates used in this study were obtained from the root nodules of soybean harvested from Da in the Nadowli District of Upper West Region, Ghana. The Upper West Region lies within the Guinea savanna agroecological zone and is characterised by grassland and savanna vegetation. It is a warm, semiarid environment with unimodal rainfall of 800 to 1100 mm, which commences in May and ends in October each year. The experimental field had no history of rhizobia inoculation. The plants were sampled at the flowering stage and the nodules were detached from the roots, washed with running water, placed in vials, and dried on silica gel covered with cotton wool [32].

### 2.2. Rhizobial Isolation

For bacterial isolation and purification, the nodules were rehydrated by immersing them in distilled water for 2 h. The nodules were then surface-sterilised by exposing them to ethanol (75%) for 10 s, followed by washing in sodium hypochlorite (3%) for 2 min, and rinsed five times with sterilised distilled water. The nodules were then crushed in sterile petri dishes with a drop of autoclave water using a glass rod [32,33], and the nodule suspension was streaked onto sterile yeast mannitol agar (YMA) plates, and incubated at 28 ± 2 °C for 5 to 12 days. Daily observations were made for the appearance of single rhizobial colonies. Bacterial colonies were purified by re-streaking on YMA plates until pure single colonies were obtained. Stock cultures of the single colonies were maintained in 50% glycerol-YMB at −80 °C for long-term use [33].

### 2.3. Authentication of Rhizobial Isolates

**Figure 1:**
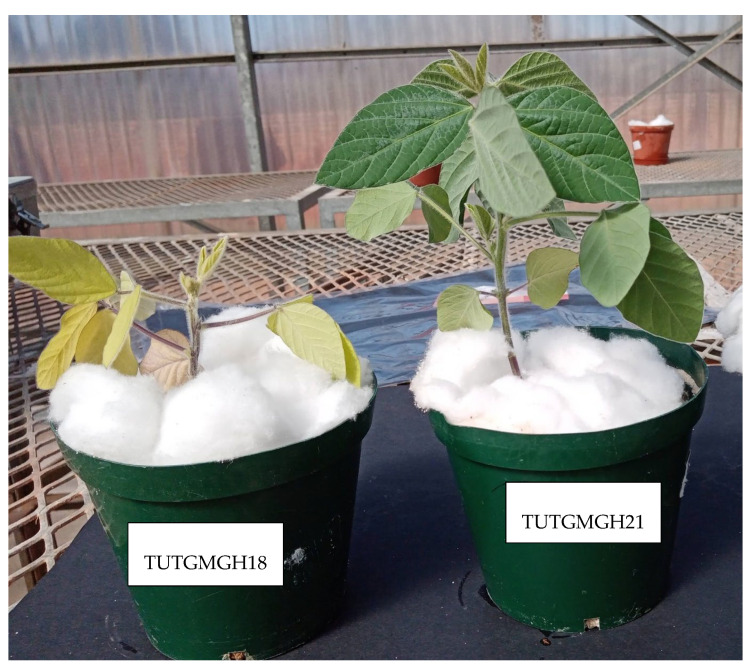
Photo of inoculated soybean plants showing greater symbiotic effectiveness and dark leaves with isolate TUTGMGH21, compared to pale leaves with ineffective isolate TUTGMGH18.

Single colonies of the bacteria were tested for their ability to induce nodule formation in their homologous host using soybean cv. Favour. Prior to planting, river sand was autoclaved in clean, washed pots, and seeds surface-sterilised by soaking in 95% ethanol for 10 s, then in sodium hypochlorite (3%) for 3 min, followed by rinsing six times in sterile distilled water [33]. Two seeds were sown per pot, and three replicated pots were used for each isolate. The pots were arranged in a randomised complete block design in the glasshouse at the Tshwane University of Technology. The glasshouse was naturally lit, with an uncontrolled temperature. The mean daily temperature during the experiment was 28 °C. After germination, the seedlings were thinned out to one plant per pot and then inoculated with bacterial cultures seven days after germination, using 1 mL per plant of rhizobial culture grown in yeast mannitol broth (YMB) to exponential phase (1 × 10^9^ cells mL^−1^). The commercial *Bradyrhizobium* strain WB74 obtained from Soygro, Potchefstroom, South Africa, was used as a positive control. Plants receiving 5 mM potassium nitrate (KNO_3_) every week, and uninoculated plants were included as additional controls. The soybean seedlings were irrigated with sterile N-free nutrient solution [32,33] and deionised water, when necessary. The soybean plants were harvested eight weeks after planting, and root nodulation was assessed. A dark leaf colour (Figure 1) and pink nodule internal colouration indicated effective nodulation.

### 2.4. Characterization of Rhizobial Isolates

The 31 authenticated isolates were all able to elicit nodulation in the homologous soybean host. The colony morphology and the appearance of the authenticated rhizobial isolates were assessed by re-streaking on YMA media and incubation at 28 °C for 5–12 days. The number of days taken for colonies to appear was used to classify the bacteria into three groups: fast-growers (<3 days), intermediate-growers (3–5 days), and slow-growers (≥6 days). The colony colour was recorded as milkish or white, and the texture was described as watery, gummy, or dry. The colony shape was characterised as circular or irregular, and the elevation was recorded as convex or flat. The colony size was measured as the colony diameter, using graph paper to the nearest millimetre [32,34].

### 2.5. Leaf Gas-Exchange Studies

Photosynthetic measurements were made on three fully expanded young trifoliate leaves per plant for three replicate plots between 08:00 and 11:00 at 60 days after planting (DAP) using a portable infrared gas analyser, version 6.2 (Li-6400XT, Li-COR, Lincoln, NE, USA). The leaves were allowed to acclimatise to the light environment in the chamber for 4 to 5 min before each measurement was taken. The instrument was calibrated to maintain the following conditions in the leaf chamber before use: a light intensity of 1200 μmol photons m^−2^s^−1^, a reference CO_2_ concentration of 400 ppm, a flow rate of 400 μmol s^−1^, a leaf temperature of 25 °C, and a relative humidity of 44%. The gas-exchange parameters measured included net photosynthesis (A), transpiration (E), stomatal conductance (gs), intercellular CO_2_ concentration (Ci), and the ratio of intercellular CO_2_ to ambient CO_2_ concentration (Ci/Ca). The intrinsic water-use efficiency (WUEi) was calculated as the ratio of A to gs [35,36].

### 2.6. Assessing Relative Symbiotic Effectiveness of Rhizobial Isolates

The effectiveness of the rhizobial isolates was determined at 60 days after planting. The harvested plants were separated into shoots, roots, and nodules. The strain symbiotic efficacy isolates were measured as nodule number per plant and nodule fresh weight per plant. The plant shoots and roots were separated, oven-dried separately at 60 °C for 48 h, and weighed. The percent relative symbiotic effectiveness (%RSE) of rhizobial isolates was calculated by expressing the shoot dry matter of soybean plants inoculated with the test isolates as a percentage of the shoot dry matter of plants inoculated with the commercial *Bradyrhizobium* inoculant strain WB74, as described in earlier studies [37,38,39], as follows:

(1) $$\%\mathrm{RSE}=\frac{Shoot\;dry\;matter\;of\;plants\;inoculated\;with\;test\;isolates}{shoot\;dry\;matter\;of\;plants\;inoculated\;with\;Bradyrhizobium\;sp.WB74}\times 100$$

The isolates were considered ineffective at <50% RSE, moderately effective at 50 to 80% RSE, and highly effective at >80% RSE.

### 2.7. Shoot ^15^N/^14^N and ^13^C/^12^C Isotopic Analysis

To assess N_2_ fixation and C assimilation in the test soybean plants, the oven-dried shoot samples were analysed for their ^15^N/^14^N and ^13^C/^12^C isotopic composition using a mass spectrometer at the Stable Light Isotope Laboratory, University of Cape Town, South Africa. Briefly, about 2 to 3 mg of ground plant samples were weighed into aluminium capsules and fed into a Carlo Erba NA1500 elemental analyser (Fisons Instruments SpA, Strada, Rivoltana, Milan, Italy) coupled to a Finnigan MAT252 mass spectrometer (Fisons Instrument SpA, Strada, Rivoltana, Milan, Italy) via conflo II open-split device to measure ^15^N/^14^N isotopic composition. A standard (Merck Gel: δ^15^N = 6.8‰, N% = 14.64) was included together with a blank sample and run after every 12 samples to calibrate the machine and avoid errors during the isotopic fractionation. All the results were referenced to air for the N isotope values. The isotopic composition (δ^15^N) was calculated as [40] follows:

(2) $$\delta^{15}N\;\left(‰\right)=\frac{\;[15_{N}/14_{N}{]}_{\mathrm{sample}}-{\left[15_{N}/14_{N}\right]}_{\mathrm{atm}}}{{\left[15_{N}/14_{N}\right]}_{\mathrm{atm}}}\;\times \;1000$$

The ^13^C natural abundance, or δ^13^C (‰), was also calculated as [41] follows:

(3) $$\delta^{13}C\left(‰\right)=\frac{[{13_{C}/12_{C}]}_{\mathrm{sample}}-{\left[13_{C}/12_{C}\right]}_{\mathrm{standard}}}{{\left[13_{C}/12_{C}\right]}_{\mathrm{standard}}}\times \;1000$$

The C and N content of the soybean shoots were calculated as the product of the shoot biomass and percent C concentration (%C) or percent N concentration (%N) [42]. The %C and %N were obtained directly from the mass spectrometer. The shoot N-fixed was calculated as follows:

N-fixed = shoot N content (nodulated plants) − shoot N content (uninoculated plants)(4)

### 2.8. Physiological Characterisation of Isolates

#### 2.8.1. Assessing Temperature Tolerance

To assess the temperature tolerance of the rhizobial isolates, 10 μL of single-colony culture was pipetted onto YMA plates and incubated at 25, 28, 30, 37, 40, and 45 °C. The tolerance of each isolate was evaluated by observing the growth of colonies on the plates for up to seven days, as described by Mohammed et al. [43].

#### 2.8.2. Measuring Drought Tolerance

The tolerance of isolates to drought was determined using yeast mannitol broth containing polyethylene glycol (PEG-6000) at 5, 15, and 30% concentrations (*w*/*v*). For this, a 10 μL volume of the test rhizobial culture was pipetted onto YMA plates that were supplemented with the different levels of PEG-6000, and incubated at 28 °C for 72 h. The isolates were shaken on a daily basis and the growth was measured at a wavelength of 600 nm using a spectrophotometer (SpectraMax® 190 microplate reader, Molecular Devices, Sunnyvale, CA, USA). The optical density (OD) values of the test isolates were used as a measure of their tolerance to drought. Thus, an OD < 0.30 was considered to be highly sensitive to drought; an OD = 0.30–0.39 as sensitive to drought; an OD = 0.40–0.50 as tolerant; and an OD > 0.5 as highly tolerant to drought [44].

#### 2.8.3. Determining Salinity Tolerance

The ability of rhizobial isolates to form colonies in the presence of different salt concentrations was assessed. Yeast mannitol agar was supplemented with different levels of NaCl at 0.05, 2.5, 5.0, 10.0, 15.0, 20.0, and 25.0 g per 500 mL YMA, equivalent to 0.01, 0.5, 1, 2, 3, 4, and 5%. This was followed by pipetting a 10 μL volume of the test rhizobial culture onto each plate and incubation at 28 °C for seven days. The bacterial growth rates were scored as +++ indicating full growth, ++ moderate growth, + weak growth, and − no growth [43].

#### 2.8.4. Assessing pH Tolerance

To assess the ability of the test isolates to tolerate different pH levels (pH 4.5, 5, 6, 7, and 8.5), five yeast mannitol broth cultures were prepared with different pH levels in 200 mL Erlenmeyer flasks [45]. For pH values less than 7, an MES hydrate buffer (2-(N-morpholino)ethanesulfonic acid) (Ssigma-Aldrich, Burlington MA, USA) was added to the flask, and for pH 7 and 8.5, HEPES (4-(2-hydroxyethyl)-1-pipera zineethanesulfonic acid (Merck, Burlington MA, USA) was added to the flask containing the broth culture. A 10 µL volume of test isolate was added into the media, adjusted to each pH level, and incubated for seven days at 28 °C. Rhizobial growth was measured at 660 nm using a spectrophotometer (SpectraMax® 190 microplate reader, Molecular Devices, Sunnyvale, CA, USA).

#### 2.8.5. Acid–Alkali Production

The isolates were cultured on YMA plates containing 0.025 g per litre of Bromothymol blue (BTB), which was used as the acid and base indicator, and incubated at 28 °C for seven days [32]. The colony formation and colour change of the medium were observed for 2–7 days. Fast-growing isolates usually change the medium to yellow due to acid production, and slow-growers to blue.

### 2.9. Statistical Analysis

All data were tested for normality and then subjected to a one-way analysis of variance using Statistica software (version 10.1). Where treatments showed significant differences, the means were separated using Duncan’s multiple range test at *p* ≤ 0.05. Correlation and regression analyses were performed to assess the existence of any relationships between the measured parameters.

## 3. Results

### 3.1. Gas-Exchange Parameters

At 60 days after planting, gas-exchange measurements were taken on the leaves of soybean seedlings nodulated by the 31 test isolates to assess differences in photosynthesis, which indirectly reflects the symbiotic functioning of the isolates used to inoculate the plants (Table 1). The rate of photosynthesis was highest in soybean plants inoculated with isolates TUTGMGH11 (16.18 µmol CO_2_ m^−2^s^−1^) and TUTGMGH8 (16.16 µmol CO_2_ m^−2^s^−1^) (Table 1). The uninoculated control plants induced the lowest photosynthetic rates (2.64 µmol CO_2_ m^−2^s^−1^). Of the 31 test isolates, 16% elicited higher photosynthetic rates (14.71–16.18 µmol CO_2_ m^−2^s^−1^) than the NO_3_-fed plants (14.47 µmol CO_2_ m^−2^s^−1^). Generally, the isolates that showed greater stomatal conductance also elicited greater CO_2_ uptake via photosynthesis. For example, the plants inoculated with isolates TUTGMGH8, TUTGMGH11, TUTGMGH22, and TUTGMGH4, and the commercial *Bradyrhizobium* strain WB74, all showed an increase in stomatal conductance and, hence, greater photosynthetic rates (Table 1).

More than 40% of the isolates induced higher intercellular CO_2_ concentrations than the commercial strain *Bradyrhizobium* strain WB74. Isolates TUTGMGH19 and TUTGMGH20 elicited the highest intercellular CO_2_ concentrations in the host soybean when compared to the other test isolates. In contrast, isolates TUTGMGH21 and TUTGMGH26 caused lower levels of intercellular CO_2_ (230.75 and 209.91 µmol CO_2_ molair^−1^, respectively). About 28 isolates also elicited higher intercellular CO_2_ concentrations in the soybeans than in the NO_3_-fed plants (Table 1).

Greater leaf transpiration rates were generally associated with increased photosynthetic rates and higher stomatal conductance. For example, isolate TUTGMGH8 and the commercial *Bradyrhizobium* strain WB74, as well as TUTGMGH4, induced the highest leaf transpiration (12.62, 12.12, 10.11, and 9.09 mol H_2_O m^−2^s^−1^, respectively), much higher photosynthetic rates, and greater stomatal conductance (Table 1). Including the commercial *Bradyrhizobium* strain WB74, 16% of the isolates induced greater leaf transpiration than the NO_3_-fed plants. As expected, the uninoculated control plants showed the lowest leaf transpiration rates, stomatal conductance, and photosynthetic rates (Table 1).

The ratio of the intercellular to ambient CO_2_ concentrations also differed significantly, with values ranging from 0.53 in plants inoculated with isolate TUTGMGH26 to 12.10 in soybean plants inoculated with the commercial *Bradyrhizobium* strain WB74 (Table 1). Isolates TUTGMGH6, TUTGMGH8, TUTGMGH11, TUTGMGH17, TUTGMGH20, and TUTGMGH23 elicited significantly higher intercellular to ambient CO_2_ concentrations than the commercial strain *Bradyrhizobium* strain WB74 (0.75). However, 21 isolates induced greater leaf intercellular to ambient CO_2_ concentrations than the commercial *Bradyrhizobium* strain WB74.

The soybean plants inoculated with isolate TUTGMGH26 recorded the highest intrinsic water-use efficiency (97.09 µmol CO_2_ mol^−1^ H_2_O), followed by the plants inoculated with isolates TUTGMG7H30, TUTGMGH25, and TUTGMGH15 (71.43–72.20 µmol CO_2_ mol^−1^ H_2_O) (Table 1). About 81% of the isolates induced higher intrinsic water-use efficiency than the commercial *Bradyrhizobium* strain WB74, while 19% induced greater intrinsic water-use efficiency than plants receiving 5 mM KNO_3_. As to be expected, plants that showed lower photosynthetic rates, stomatal conductance, leaf transpiration, and intercellular CO_2_ concentrations induced much higher intrinsic water-use efficiency, except for the uninoculated control plants (Table 1).

### 3.2. Plant Growth

The shoot biomass of soybean plants inoculated with the 31 rhizobial isolates ranged from 0.45 to 1.99 g plant^−1^ (Table 2). The highest shoot biomass was induced by isolate TUTGMGH21 (1.99 g plant^−1^), followed by the 5 mM NO_3_-fed plants (1.85 g plant^−1^), and isolate TUTGMGH3 (1.74 g plant^−1^). High leaf photosynthesis was generally associated with an increased shoot biomass and vice versa. For example, isolates TUTGMGH14, TUTGMGH18, and TUTGMGH26, which induced lower photosynthetic rates, resulted in the lowest shoot biomass (0.45–0.72 g plant^−1^), followed by the uninoculated control plants (0.33 g plant^−1^) (Table 2).

The root biomass varied significantly among the plants inoculated with the test isolates, with TUTGMGH6, TUTGMGH21, and TUTGMGH30 inducing the highest root dry matter accumulation (0.72–0.85 g plant^−1^). However, the highest root dry matter was recorded by the 5 mM NO_3_-fed plants (1.13 g plant^−1^), and the lowest by the uninoculated control (0.22 g plant^−1^ (Table 2).

The whole-plant biomass (shoot + root) differed significantly (*p* ≤ 0.001), with 5 mM NO_3_-fed plants producing the highest total plant biomass (2.97 g plant^−1^), followed by plants inoculated with isolates TUTGMGH1, TUTGMGH3, TUTGMGH6, TUTGMGH9, TUTGMGH20, TUTGMGH21, and TUTGMGH30 (2.23–2.70 g plant^−1^). About 77% of the rhizobial isolates induced greater accumulation of whole plant biomass (1.69–2.70 g plant^−1^) than the commercial *Bradyrhizobium* strain WB74 (Table 2).

### 3.3. Shoot C Concentration

The C concentration (%C) of the soybean shoots varied significantly (*p* ≤ 0.01) with inoculation and ranged from 40.15% to 44.42% (Table 2). Isolates TUTGMGH24 and TUTGMGH29 elicited the highest shoot C concentrations (44.42 and 44.30%, respectively), followed by the commercial *Bradyrhizobium* strain WB74, TUTGMGH28, TUTGMGH30, and TUTGMGH20. The lowest shoot C concentration (40.15%) was recorded in the uninoculated control plants.

In general, higher shoot biomass was associated with increased shoot C content and accumulation. As a result, the 5 mM NO_3_-fed plants and the plants inoculated with isolates TUTGMGH1, TUTGMGH3, TUTGMGH20, TUTGMGH23, TUTGMGH25, TUTGMGH21, and TUTGMGH24 recorded greater shoot biomass, and, hence, higher shoot C content. In contrast, the plants inoculated with isolates TUTGMGH10, TUTGMGH14, TUTGMGH18, TUTGMGH22, TUTGMGH26 and the uninoculated control recorded the least shoot biomass and, hence, the lowest shoot C content. About 81% of the isolates induced greater shoot C content (51.86–84.98 g plant^−1^) than the commercial *Bradyrhizobium* strain WB74 (42%) (Table 2).

### 3.4. Shoot δ^13^C and C:N Ratio

The shoot δ^13^C values of the soybean plants differed significantly (*p* ≤ 0.01) with the test isolates, ranging from −29.55‰ to −27.19‰. Inoculating the soybean plants with isolate TUTGMGH9 resulted in greater shoot δ^13^C (−27.19‰), followed by isolates TUTGMGH11, TUTGMGH24, TUTGMGH26, and TUTGMGH27 (−27.34‰ to −27.23‰). In contrast, lower δ^13^C values were recorded in the plants inoculated with the commercial *Bradyrhizobium* strain WB74 (−29.55‰), and those inoculated with isolates TUTGMGH23 (−28.44‰) and TUTGMGH25 (−28.29‰).

The soybean plants treated with 5 mM KNO_3_ recorded the highest C:N ratio (23.59 g g^−1^), followed by the plants inoculated with the commercial *Bradyrhizobium* strain WB74 (21.59 g g^−1^) and isolate TUTGMGH13 (21.54 g g^−1^). In contrast, the plants inoculated with isolates TUTGMGH7 and TUTGMGH25 recorded the lowest C:N ratios (15.57 and 15.91 g g^−1^, respectively) (Table 2).

### 3.5. Nodulation Induced by Rhizobial Isolates

The nodule number of the soybean plants inoculated with the different rhizobial isolates varied from 10 to 43 per plant, while the nodule fresh mass ranged from 0.18 to 0.64 g plant^−1^ (Table 3). The plants inoculated with isolate TUTGMGH20 produced the most root nodules (43 nodules per plant), followed by isolates TUTGMGH3 and TUTGMGH25, which produced similarly high nodule numbers (37 and 35 nodules per plant, respectively). Of the 31 isolates tested, 16% induced significantly greater nodule numbers on the soybeans than the commercial *Bradyrhizobium* strain WB74 (which formed 24 nodules per plant), while about 71% of them induced fewer nodules in the test soybeans than the commercial *Bradyrhizobium* strain WB74. Higher nodule numbers generally correlated with greater nodule fresh weights, though there were a few exceptions due to differences in nodule size. For example, isolates TUTGMGH4, TUTGMGH9, TUTGMGH12, and TUTGMGH25 induced more nodules in the soybeans, resulting in the highest nodule fresh weights (0.51–0.6 g plant^−1^). However, despite inducing fewer nodule numbers per plant, isolates TUTGMGH1, TUTGMGH2, and TUTGMGH6 elicited relatively high nodule fresh masses in the soybeans due to bigger nodule sizes. About 90% of the isolates produced greater nodule biomass (0.31– 0.64 g plant^−1^) than the commercial *Bradyrhizobium* strain WB74. In contrast, isolates TUTGMGH26 and TUTGMGH27 induced much lower nodule numbers (16 nodules per plant), and, hence, small nodule fresh weights (0.18 and 0.24 g plant^−1^) (Table 3).

### 3.6. Relative Symbiotic Effectiveness of Rhizobial Isolates

**Figure 2:**
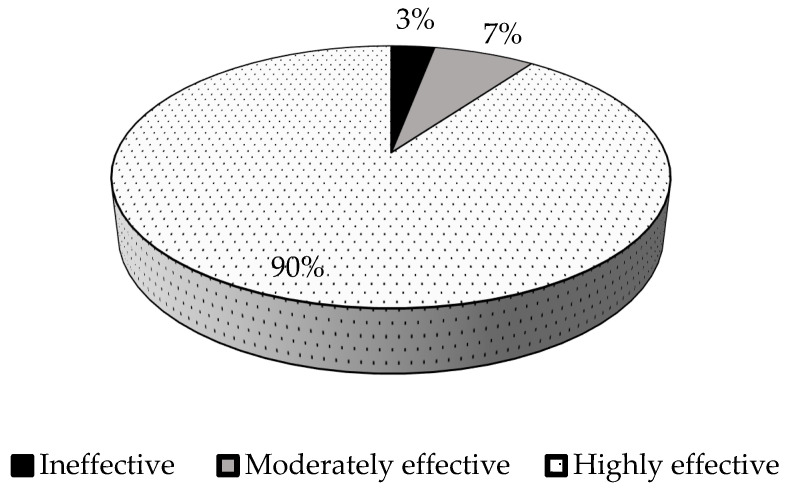
Classification of 31 native rhizobial isolates based on percent relative symbiotic effectiveness.

The percent relative symbiotic effectiveness varied significantly (*p* ≤ 0.05) among the test isolates, with a range of 42% to 186% (Table 3; Figure 2). According to Rejili et al. [46], the isolates with less than 50% RSE are considered ineffective, 50–80% as moderately effective, and greater than 80% as highly effective. In this study, the isolates were effective, except isolate TUTGMGH14, which scored 42% relative symbiotic effectiveness. Isolates TUTGMGH18 and TUTGMGH26 were moderately effective with 67% and 56% RSE, while the rest of the isolates (90%) were highly effective (%RSE ≥ 80%) (Figure 2). Furthermore, 75% of the isolates were more effective than the commercial *Bradyrhizobium* strain WB74. Isolate TUTGMGH21 was the most effective, with 186% RSE (Table 3).

### 3.7. Shoot N Concentration

Inoculating soybeans with rhizobial isolate TUTGMGH23 resulted in a significantly greater shoot N concentration (2.92%), followed by isolates TUTGMGH19, TUTGMG7, and TUTGMGH16 (Table 3). Quite expected, the lowest shoot N concentration (1.13%) was recorded in the uninoculated control plants. However, the plants inoculated with isolates TUTGMGH23 and TUTGMGH21 produced the highest shoot N content, with the uninoculated control (0.37 g plant^−1^) and the plants inoculated with isolates TUTGMGH18 and TUTGMGH26 recording the lowest shoot N content (1.64 and 1.57 g plant^−1^) (Table 3).

### 3.8. Shoot δ^15^N and N-Fixed

The highest shoot δ^15^N values were recorded in the uninoculated control plants (+2.16‰), followed by the 5 mM KNO_3_-fed plants (+1.55‰) (Table 3). Isolates TUTGMGH24 (−1.26‰) and TUTGMGH14 (−1.26‰) recorded the highest δ^15^N values. The remaining isolates recorded much lower shoot δ^15^N values, which ranged from −2.64‰ to −1.76‰ (Table 3). The amount of N-fixed by the isolates in the soybeans differed significantly, with values ranging from 0.75 g plant^−1^ with isolate TUTGMGH14 to 4.77 g plant^−1^ with isolate TUTGMGH21 (Table 3).

### 3.9. Phenotypic Characterisation of the Rhizobial Isolates

**Figure 3:**
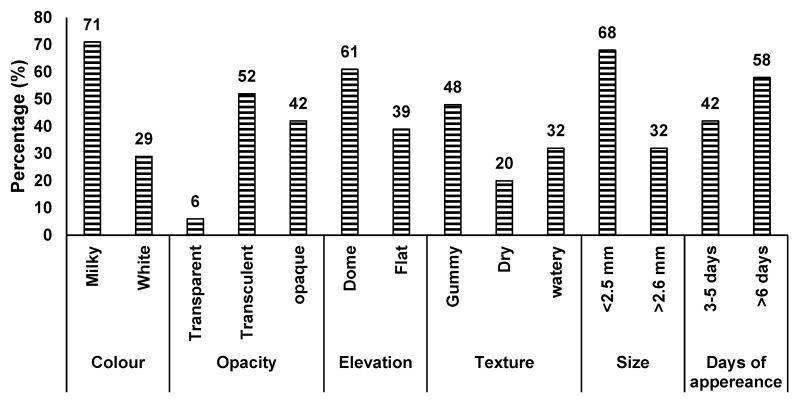
Phenotypic characterisation of 31 soybean rhizobial isolates.

A total of 31 authenticated soybean rhizobial isolates were evaluated for phenotypic characteristics, such as colony colour, size, texture, elevation, opacity, shape, and the number of days to colony appearance on YMA plates (Figure 3 and Tables S1 and S2). Of the 31 isolates, 42% were classified as intermediate-growers (3–5 days to appear on YMA plates) and 58% as slow-growers (6–8 days). In terms of the colony colour, 22 isolates (71%) were milkish, while the remaining 9 isolates (29%) were white. Regarding colony elevation, 19 isolates (61%) were convex, and 12 isolates (39%) were flat. A number of the isolates were gummy (48%), or watery (32%), and 6 isolates (20%) showed a dry texture. About 94% of the isolates had a circular shape, with only two isolates being irregular. Two isolates (TUTGMGH15 and TUTGMGH23) were transparent, 16 isolates translucent, and the remaining 13 isolates opaque. Most isolates had small colonies with diameters less than 2.5 mm (68%), while the remainder had diameters between 2.5 and 4 mm (Figure 3 and Figure S1). The isolates were also cultured in YMA medium containing Bromothymol Blue (BTB) as the acid/base indicator, and 45% of the isolates turned the medium into yellow within 5 days of incubation, while the remaining isolates turned blue.

### 3.10. Biochemical Characterisation of Rhizobial Isolates

#### 3.10.1. Temperature Tolerance

Most of the test isolates showed growth at all tested temperatures, except TUTGMGH5, TUTGMGH9, and TUTGMGH28, which failed to grow at 40 and 45 °C (Table 4). Isolates TUTGMGH3 and TUTGMGH4 showed weak growth at all the temperature levels. The test isolates generally exhibited maximum growth at temperatures ranging from 28 to 30 °C. At 25 °C, 14 isolates (45%) showed weak growth, but 6 isolates showed full growth. Only isolate TUTGMGH17 showed good growth at all the temperature levels tested (Table 4).

#### 3.10.2. Salinity Tolerance

All the isolates showed growth in media supplemented with 0.01% (control) and 0.5% NaCl (Table 4). However, isolates TUTGMGH5, TUTGMGH11, and TUTGMGH16 grew in only the 0.01% control but were susceptible to 0.5 to 5% NaCl (Table 4). In contrast, isolates TUTGMGH8, TUTGMGH22, and TUTGMGH27 (in that order), showed moderate to full cell growth at all the salinity levels tested (Figure S2). However, it was only isolate TUTGMGH8 that showed full growth at all the salinity levels (0.01–5% NaCl), followed by TUTGMGH3, TUTGMGH7, TUTGMGH9, TUTGMGH22, TUTGMGH27, and TUTGMGH31, which showed moderate growth at 5% NaCl (Table 4).

#### 3.10.3. Drought Tolerance

Drought tolerance was evaluated in the soybean isolates using polyethylene glycol (PEG)-6000 at different levels, ranging from 5 to 15 to 30% (Table 5). There was a significant suppression of rhizobial cell growth at 15 and 30% PEG-6000. Although isolates TUTGMGH12 (0.424) and TUTGMGH19 (0.461) could tolerate 5% PEG-6000, TUTGMGH9 (0.523) was highly tolerant of PEG-6000, with the rest showing significant cell growth inhibition at 5% PEG-6000. Of all the 31 isolates tested, TUTGMGH12 showed better cell growth at 5, 15, and 30% PEG-6000 than the other isolates, followed by TUTGMGH10, TUTGMGH16, and TUTGMGH15 (Table 5). The isolates that were intolerant of 30% PEG included TUTGMGH17 and TUTGMGH29.

#### 3.10.4. IAA Production

The rhizobial isolates differed significantly (*p* ≤ 0.05) in their ability to produce IAA using Salkowski’s reagent. The maximum IAA production was by isolate TUTGMGH15 (11.37 µg mL^−1^), followed by TUTGMGH3, TUTGMGH8, and TUTGMGH5 (9.92, 9.85, and 9.59 µg mL^−1^). Isolates TUTGMGH26 (0.98 µg mL^−1^) and TUTGMGH24 (1.21 µg mL^−1^) produced the lowest IAA (Table 5).

#### 3.10.5. pH Tolerance

The 31 rhizobial isolates exhibited varied responses to different pH levels. The maximum growth in most of the isolates was recorded at pH 8.5, with the lowest at pH 6 (Table 6). In this study, 21 out of the 31 isolates recorded maximum growth at pH 8.5, and these included TUTGMGH4, TUTGMGH9, TUTGMGH10, TUTGMGH12, TUTGMGH17, TUTGMGH18, TUTGMGH21, TUTGMGH21, TUTGMGH30, and TUTGMGH31. However, some isolates, such as TUTGMGH4, TUTGMGH9, and TUTGMGH12, tolerated a wider range of pH conditions, ranging from acidic to alkaline.

## 4. Discussion

### 4.1. Diversity of Soybean Rhizobial Isolates and Their Photosynthetic Performance

The presence of diverse rhizobial populations in soils represents an opportunity to identify effective strains for inoculant formulation [47]. With a changing climate, however, the identified rhizobia should have multiple traits for survival in a changing environment that is characterised by high-temperature extremes, drought, salinity, low pH, and low soil nutrient concentrations. The soybean rhizobia obtained from Da in the Upper West Region of Ghana exhibited diverse characteristics. Single colonies isolated from the soybean root nodules displayed a circular shape (>94%) and milkish colour (>71%), as well as a gummy or watery texture (>48 and 32% respectively), were small in size, with diameters of less than 2.5 mm (>68%), and consisted of slow-growers (58%) and intermediate-growers (42%). These colony characteristics suggest that the soybean rhizobia from Da belong to the genus *Bradyrhizobium*, as described by Somasegaran and Hoben [32] and Pongslip [34]. *Bradyrhizobium* is the most dominant rhizobial microsymbiont in Africa [48,49,50,51], and is the primary nodulator of soybeans in Ghana [6].

Because bacteroids in legume root nodules require de novo photosynthate to reduce N_2_ to NH_3_, photosynthetic rates during legume plant growth generally correlate with the symbiotic efficacy of the nodule occupants [30,52]. In this study, gas-exchange measurements revealed marked differences in photosynthetic functioning, stomatal conductance, leaf transpiration, and water-use efficiency in soybean plants nodulated by the 31 rhizobial isolates from Da, Ghana. In fact, isolates TUTGMGH4, TUTGMGH8, TUTGMGH11, and TUTGMGH22, as well as the commercial *Bradyrhizobium* strain WB74, generally induced greater photosynthetic rates, powered by higher stomatal conductance, which permitted greater CO_2_ influx into photosynthetic cells. In contrast, the soybean plants nodulated by isolates TUTGMGH14, TUTGMGH18, and TUTGMGH26 revealed low photosynthesis due to reduced stomatal conductance that led to reduced CO_2_ influx and, hence, limited accumulation of shoot and whole-plant biomass.

In this study, there was no link between increased nodulation and plant biomass accumulation for some isolates, as reported previously [53]. As a result, although isolatesTUTGMGH8, TUTGMGH11, TUTGMGH22, and TUTGMGH30, and the commercial *Bradyrhizobium* strain WB74 elicited higher photosynthetic rates, they showed lower nodule dry matter. This contrasted with the lower photosynthetic rates, stomatal conductance, and leaf transpiration induced by isolates TUTGMGH1, TUTGMGH3, and TUTGMGH21, which recorded much higher nodulation, shoot dry matter, and shoot C concentrations (Table 1, Table 2 and Table 3). The instantaneous nature of gas-exchange measurements implies that they are strongly influenced by environmental factors, and this could account for the observed discrepancy [35].

C accumulation in legumes is a function of photosynthesis, which is dependent on symbiotic N_2_ fixation for the biosynthesis of the light-harvesting chlorophyll molecules and the CO_2_-reducing Rubisco enzyme. It was, therefore, not surprising that soybean inoculated with rhizobial isolates in this study increased C accumulation, compared to 5 mM NO_3_ -feeding, with values ranging from 40.15 to 44.30 g plant^−1^. It was also interesting to note that the rhizobial isolates that induced high shoot C concentrations were associated with more negative shoot δ^13^C values and vice versa, except for isolate TUTGMGH24. This implies that shoot C accumulation via photosynthesis was linked to plant water-use efficiency, as evidenced by the positive correlation between the shoot C concentration and shoot δ^13^C (r = 0.52 **) (Table S2).

### 4.2. Plant Water-Use Efficiency and Strain Symbiotic Effectiveness

Shoot δ^13^C is a known measure of water-use efficiency in C_3_ plants [41]. In this study, isolates TUTGMGH9, TUTGMGH11, TUTGMGH19, TUTGMGH24, TUTGMGH26, TUTGMGH27, and TUTGMGH30 induced greater shoot δ^13^C values (Table 2), and hence, greater water-use efficiency. However, isolates TUTGMGH26, TUTGMGH15, TUTGMGH21, TUTGMGH24, TUTGMGH31, and TUTGMGH9 also showed higher photosynthetic water-use efficiency, in addition to greater shoot δ^13^C values (Table 1 and Table 2). These results suggest that, as tools, the shoots ^13^C and photosynthetic water-use efficiency were robust enough in identifying water-use efficiency in soybeans nodulated by different rhizobial strains. Furthermore, isolates TUTGMGH9, TUTGMGH19, and TUTGMGH21 did not only exhibit greater shoot δ^13^C, but they also showed high shoot N content or N-fixed, as well as increased relative symbiotic effectiveness (Table 3). These results suggest that water-use efficiency was strongly enhanced by potent symbiotic signals that served as environmental cues, in addition to increasing N_2_ fixation and N nutrition in the legume.

However, the fact that isolates TUTGMGH7, TUTGMGH9, TUTGMGH23, and TUTGMGH25, which obtained a high proportion of their N nutrition from symbiosis and yet exhibited low water-use efficiency, could suggest that partial closure of the stomata during soil water deficit affected photosynthesis, leading to reduced water-use efficiency. This was supported by the negative correlation found between shoot δ^13^C and stomatal conductance (r = −0.28 **), as well as between shoot δ^13^C and leaf transpiration rates (r = −0.40 ***) (Table S2).

The symbiotic functioning of the 31 soybean isolates from Da varied significantly, as evidenced by the observed differences in the nodule number, nodule biomass, shoot N concentration, shoot δ^15^N, amount of N-fixed, and shoot N content. The marked differences in soybean biomass were generally linked to isolate symbiotic efficacy. For example, isolates TUTGMGH16, TUTGMGH21, TUTGMGH23, and TUTGMGH25, which were among the isolates that induced the highest nodulation (nodule number and weight), showed the lowest δ^15^N values and exhibited greater shoot N concentrations and content, as well as the highest amount of N-fixed, producing the largest soybean biomass (Table 2 and Table 3). Metabolites such as lumichrome and riboflavin, produced by rhizobia during nodule formation, serve as environmental cues for sensing soil moisture deficit [54,55,56]. High N_2_-fixing rhizobia apparently release more lumichrome than low-fixing microbes [57], suggesting a link between rhizobial strain effectiveness, symbiotic N nutrition, and legume water-use efficiency. It is not surprising that highly effective isolates, such as TUTGMGH9, TUTGMGH19, and TUTGGMGH21, showed high amounts of N-fixed and greater shoot δ^13^C or water-use efficiency in this study. The results of this study suggest that an increase in nodule functioning induced by the test isolates increased C and N assimilation through photosynthesis and N_2_ fixation, leading to greater biomass accumulation. In contrast, isolates TUTGMGH14, TUTGMGH18, and TUTGMGH26 produced the lowest nodule mass and N concentrations, high shoot δ^15^N, and low amounts of N-fixed induced least shoot biomass. These results were supported by the significant correlations between the shoot dry matter and nodule biomass (r = 0.43 ***) and shoot N concentration (r = 0.31 ***) (Table S2). These findings clearly indicate that soybean dry matter accumulation is directly linked to N_2_ fixation [52]. This is an argument that is consistent with the observed relationship between root nodulation and shoot biomass in Jack beans [39] and cowpeas [14]. Furthermore, the isolates that produced greater symbiotic N also recorded much higher relative symbiotic effectiveness, producing greater symbiotic N.

The data for the percent relative symbiotic effectiveness of the isolates in this study differed significantly (*p* ≤ 0.01), with a range of 42% for isolate TUTGMGH14 to 186% for isolate TUTGMGH21. Of the 31 isolates evaluated, 28 were more effective than the commercial *Bradyrhizobium* strain WB74, suggesting the presence of highly effective rhizobial populations in Ghanaian soils, with potential for use as inoculants. These isolates should, however, be further assessed for their ability to increase soybean plant growth and yield under field conditions.

### 4.3. Plant Growth-Promoting Traits of the Rhizobial Isolates from Da

Many abiotic factors can affect rhizobial growth and survival in soils. Temperature stress can alter the permeability of bacterial membranes and cause the denaturation of enzymes, leading to cell death and low rhizobial populations [58]. Under laboratory conditions, the growth in the test isolates was not markedly affected by temperature, except for TUTGMGH5, TUTGMGH9, and TUTGMGH28, which failed to grow at temperatures above 40 °C. The ability of these isolates to grow in a wide range of temperatures gives them a competitive edge in the rhizosphere to survive and nodulate their host plants. However, isolates are generally origin-related, and therefore, rhizobia from hot climates may generally tolerate high temperatures and vice versa. Yuan et al. [25], for example, found greater shoot biomass and N_2_ fixation under cooler conditions following inoculation with a cold-tolerant strain (4 °C) than the control commercial inoculant. However, Yuan et al. [25] also showed that rhizobia isolated from soybeans in hot environments could tolerate temperatures up to 44 °C, suggesting the development of heat tolerance mechanisms, such as the production of heat-shock proteins by the rhizobia for cellular protection against high temperatures [24].

The rhizobial isolates from this study were also found to be sensitive to drought, with only a few showing positive growth at 5% PEG-6000. Although isolates TUTGMGH9, TUTGMGH12, TUTGMGH19, TUTGMGH3, TUTGMGH10, and TUTGMGH30 grew well at a 5% PEG-6000 concentration, only TUTGMGH12 showed growth at 5, 15, and 30% PEG-6000, followed by TUTGMGH10, TUTGMGH16, and TUTGMGH15. Many of these isolates could, however, also induce high water-use efficiency and N_2_ fixation in soybean plants (Table 1 and Table 3), thus making them ideal for use in a changing climate where drought is frequent. The reported high nodulation of *Phaseolus vulgaris* inoculated with drought-tolerant rhizobia under conditions of soil moisture deficit [59] implies that the soybean isolates with drought tolerance could be recommended to farmers. These drought-tolerant rhizobia can apparently adjust their cell wall elasticity to prevent mechanical damage to the plasma membrane, thus improving water-use efficiency [26].

With climate change, irrigated agriculture and, hence, soil salinity are likely to increase. This would require identifying salt-tolerant crops and rhizobia for achieving food security. The 31 soybean isolates tested in this study, showed markedly different growth rates to various concentrations of salinity (Table 4), with 16 isolates showing an ability to grow at 5% NaCl, a finding better than the report by Khaitov et al. [23]. Khaitov et al. [23] identified rhizobial strains from chickpeas that had good growth at 3% NaCl. In fact, in this study, isolate TUTGMGH8 exhibited maximum growth from 0.01% to 5% NaCl and is therefore an ideal candidate for inoculant formation as a biofertiliser for soybean production under saline conditions. We also found that in this study, the slow-growing rhizobial isolates were sensitive to salinity, whereas the acid-producers could tolerate up to 5% NaCl. While 17 isolates showed low tolerance to increasing NaCl concentrations, 16 isolates were able to grow at all levels of salinity and temperature, though they differed in their growth rates (Table S1).

This study also revealed differences in isolate growth rates at various pH levels. Although these rhizobia were isolated from acidic soil, only isolates TUTGMGH3, TUTGMGH4, and TUTGMGH9 showed positive growth in an acidic medium, with their absorbances ranging from 0.479 to 0.697. This could be because the activity of H^+^ ions in the culture medium is different from that in the soil, where the charges of the colloids can partially neutralise the activity of the ions [60]. However, isolates TUTGMGH6, TUTGMGH8, TUTGMGH9, TUTGMGH10, TUTGMGH12, TUTGMGH17, TUTGMGH18, TUTGMGH21, TUTGMGH27, and TUTGMGH30 performed better under alkaline conditions. The acid-tolerant isolates were intermediate and slow-growing and changed the YMA medium supplemented with BTB into a blue colour, indicating alkaline production. Slow-growing rhizobia isolates are generally considered highly tolerant of low pH, suggesting that alkaline producers are dominant in tropical soils. In fact, Oliveira et al. [61] have suggested that alkaline-producing rhizobia are dominant in acidic soils, while acid producers are dominant in alkaline soils [62,63].

In conclusion, isolates TUTGMGH1, TUTGMGH3, TUTGMGH9, and TUTGMGH21 were best candidates for relative symbiotic effectiveness and N_2_-fixation; isolate TUTGMGH16 for drought tolerance and IAA production; isolates TUTGMGH4, TUTGMGH9, and TUTGMGH21 for pH tolerance; isolate TUTGMGH12 for drought; TUTGMGH8 for salinity tolerance and higher leaf photosynthetic activity; and TUTGMGH17 for temperature tolerance. However, for better application of these rhizobial isolates as bioinoculants, their symbiotic performance should be assessed under field conditions to ascertain their competitiveness and symbiotic efficacy.

## Figures and Tables

**Table 1:** Effect of 31 rhizobial inoculation and nitrate-feeding on leaf gas exchange in soybean plants. Values (mean ± S.E.) followed by dissimilar letters are significant at ** *p* ≤ 0.01 or *** *p* ≤ 0.001.

Isolates	A	Gs	Ci	E	Ci/Ca	WUEi
	µmol (CO_2_) m^−2^s^−1^	Mol (H_2_O) m^−2^s^−1^	µmol (CO_2_) molair^−1^	Mol (H_2_O) m^−2^s^−1^		µmol (CO_2_) mol^−1^(H_2_O)
TUTGMGH1	11.34 ± 0.76 d–l	0.16 ± 0.01 j–m	257.38 ± 1.38 fg	5.41 ± 0.07 ij	0.65 ± 0.01 i–j	70.36 ± 2.43 bc
TUTGMGH2	12.48 ± 0.40 fg	0.26 ± 0.01 d–f	285.89 ± 3.89 a–d	7.81 ± 0.21 d–h	0.74 ± 0.01 a–e	48.30 ± 2.11 d–g
TUTGMGH3	11.59 ± 0.01 g–k	0.19 ± 0.02 h–j	277.25 ± 5.98 a–f	6.12 ± 0.44 g–j	0.72 ± 0.01 a–i	63.80 ± 7.59 b–e
TUTGMGH4	14.81 ± 0.07 bc	0.33 ± 0.0001 b	285.99 ± 0.27 a–d	9.09 ± 0.01 b–d	0.75 ± 0.001 abc	44.54 ± 0.13 h–j
TUTGMGH5	10.62 ± 0.12 j–m	0.18 ± 0.03 h–l	261.91 ± 21.91 d–g	9.84 ± 2.46 cd	0.67 ± 0.06 g–j	65.69 ± 16.51 b–d
TUTGMGH6	11.81 ± 0.01 g–i	0.30 ± 0.03 c	290.18 ± 0.66 ab	7.97 ± 0.001 c–g	0.76 ± 0.003 ab	40.49 ± 3.49 ij
TUTGMGH7	11.26 ± 0.02 h–m	0.27 ± 0.003 cd	290.69 ± 3.60 ab	8.29 ± 0.004 b–f	0.75 ± 0.01 a–c	41.29 ± 0.54 ij
TUTGMGH8	16.16 ± 0.63 a	0.39 ± 0.03 a	285.08 ± 0.25 a–e	12.62 ± 0.01 a	0.76 ± 0.003 ab	41.06 ± 1.20 ij
TUTGMGH9	13.05 ± 0.10 ef	0.21 ± 0.01 g–i	266.68 ± 5.76 b–h	7.40 ± 0.04 c–h	0.70 ± 0.01 b–j	62.08 ± 2.23 b–f
TUTGMGH10	12.44 ± 0.31 fg	0.24 ± 0.01 e–g	271.05 ± 1.85 a–f	8.12 ± 0.35 c–f	0.71 ± 0.01 a–i	53.08 ± 1.38 b–i
TUTGMGH11	16.18 ± 0.01 a	0.39 ± 0.001 a	286.22 ± 0.18 a–d	10.11 ± 0.01 b	0.76 ± 0.001 ab	41.57 ± 0.11 ij
TUTGMGH12	9.03 ± 0.73 o	0.14 ± 0.002 lm	260.12 ± 9.90 e–g	6.01 ± 0.06 h–j	0.67 ± 0.02 f–j	62.60 ± 5.87 b–f
TUTGMGH13	11.78 ± 0.74 g–j	0.23 ± 0.01 e–g	274.35 ± 1.67 a–g	8.74 ± 0.10 b–e	0.70 ± 0.001 a–i	50.58 ± 0.47 e–j
TUTGMGH14	10.94 ± 0.00 h–m	0.27 ± 0.001 c–e	283.46 ± 7.89 a–e	8.38 ± 1.22 b–f	0.74 ± 0.02 a–d	40.83 ± 0.16 ij
TUTGMGH15	9.25 ± 0.04 no	0.13 ± 0.01 mn	243.343 ± 10.34 gh	7.19 ± 1.25 d–i	0.63 ± 0.02 j	71.59 ± 6.07 b
TUTGMGH16	10.15 ± 0.08 mn	0.17 ± 0.003 i–l	263.78 ± 3.02 c–g	7.09 ± 0.13 e–i	0.69 ± 0.01 c–j	58.43 ± 1.51 c–g
TUTGMGH17	10.96 ± 0.38 h–m	0.25 ± 0004 d–f	288.84 ± 3.23 a–c	8.41 ± 0.03 b–f	0.76 ± 0.01 ab	43.13 ± 1.53 ij
TUTGMGH18	7.76 ± 0.08 p	0.15 ± 0.0001 k–m	281.28 ± 0.91 a–f	7.24 ± 0.002 c–i	0.71 ± 0.002 a–i	51.77 ± 0.57–i
TUTGMGH19	11.44 ± 0.36 g–l	0.28 ± 0.003 cd	293.69 ± 2.53 a	8.67 ± 0.32 b–e	0.77 ± 0.02 a	41.01 ± 0.85 ij
TUTGMGH20	7.86 ± 0.02 p	0.21 ± 0.01 k–n	294.82 ± 3.90 a	7.84 ± 0.06 d–h	0.76 ± 0.01 ab	37.63 ± 1.68 j
TUTGMGH21	10.40 ± 0.29 lm	0.15 ± 0.002 k–m	230.75 ± 16.97 h	6.12 ± 0.002 g–j	0.61 ± 0.04 e–j	70.88 ± 2.75 bc
TUTGMGH22	15.71 ± 0.35 ab	0.30 ± 0.0014 c	272.51 ± 2.23 a–g	7.79 ± 0.11 d–h	0.71 ± 0.07 a–i	52.62 ± 0.54 e–i
TUTGMGH23	12.04 ± 0.29 f–h	0.25 ± 0.02 d–f	276.83 ± 6.81 a–f	8.34 ± 0.02 b–f	0.76 ± 0.004 ab	47.90 ± 2.15 g–j
TUTGMGH24	13.57 ± 0.42 de	0.21 ± 0.002 gh	262.06 ± 4.01 b–g	7.91 ± 0.16–h	0.67 ± 0.01 f–j	63.60 ± 1.42 b–e
TUTGMGH25	13.07 ± 0.19 ef	0.18 ± 0.002 h–k	265.45 ± 1.68 b–g	6.49 ± 0.04 f–j	0.66 ± 0.01 h–j	71.43 ± 1.26 b
TUTGMGH26	10.58 ± 0.19 j–l	0.11 ± 0.001 n	209.91 ± 3.81 i	4.80 ± 0.03 jk	0.53 ± 0.010 j	97.09 ± 2.40 a
TUTGMGH27	11.46 ± 0.01 g–l	0.20 ± 0.001 g–i	245.10 ± 20.48 gh	7.40 ± 0.54 d–h	0.64 ± 0.06 j	56.32 ± 0.23 d–h
TUTGMGH28	11.403 ± 0.08 g–l	0.22 ± 0.002 fg	279.53 ± 0.97 a–f	7.18 ± 0.05 d–i	0.73 ± 0.002 a–h	50.81 ± 0.70 f–i
TUTGMGH29	10.67 ± 0.17 i–m	0.23 ± 0.001 fg	279.44 ± 0.07 a–f	8.24 ± 0.50 b–f	0.74 ± 0.02 a–f	46.63 ± 0.63 d–j
TUTGMGH30	14.74 ± 0.71 bc	0.20 ± 0.002 g–i	259.90 ± 6.45 e–g	7.79 ± 0.47 d–h	0.64 ± 0.02 j	72.20 ± 3.08 b
TUTGMGH31	11.49 ± 0.28 g–l	0.18 ± 0.001 h–l	265.91 ± 8.97 b–g	7.68 ± 0.57 d–h	0.68 ± 0.02 d–j	64.15 ± 0.68 b–e
*Bradyrhizobium* strain WB74	15.59 ± 0.03 bc	0.37 ± 0.001 a	279.293 ± 0.24 a–f	12.10 ± 0.01 a	0.75 ± 0.001 a–d	42.39 ± 0.18 ij
Uninoculated	2.64 ± 0.26 q	0.07 ± 0.0002 o	293.67 ± 0.69 a	3.58 ± 0.01 i	0.76 ± 0.019 ab	40.43 ± 4.08 ij
5 mM KNO_3_	14.47 ± 0.53 cd	0.21 ± 0.001 g–i	256.56 ± 4.31 fg	8.81 ± 0.29 b–e	0.72 ± 0.002 a–i	70.34 ± 2.60 bc
F-statistics	59.32 **	46.96 **	6.66 ***	9.72 ***	6.99 ***	12.68 **

**Table 2:** Plant growth, shoot C accumulation, and δ^13^C of soybean inoculated with different rhizobial isolates. Values (mean ± S.E.) followed by dissimilar letters are significant at ** *p* ≤ 0.01 or *** *p* ≤ 0.001.

Isolates	Shoot Dry Matter	Root Dry Matter	Total Biomass	C Concentration	C Content	δ^13^C	C:N Ratio
	g Plant^−1^	g Plant^−1^	g Plant^−1^	%	g Plant^−1^	‰	g·g^−1^
TUTGMGH1	1.72 ± 0.09 a–d	0.64 ± 0.03 b–e	2.36 ± 0.13 bc	43.30 ± 0.05 f–h	74.48 ± 4.05 ab	−27.52 ± 0.01 e–i	18.31 ± 0.04 c–g
TUTGMGH2	1.44 ± 0.03 c–h	0.50 ± 0.05 c–g	1.94 ± 0.07 c–k	43.27 ± 0.03 f–i	62.16 ± 1.22 b–k	−27.95 ± 0.02 mn	17.99 ± 0.03 c–i
TUTGMGH3	1.74 ± 0.09 a–c	0.51 ± 0.01 c–g	2.25 ± 0.11 c–f	43.42 ± 0.28 d–g	75.58 ± 4.29 bc	−27.51 ± 0.01 d–h	18.59 ± 0.18 c–g
TUTGMGH4	1.32 ± 0.23 e–l	0.37 ± 0.01 f–h	1.69 ± 0.26 h–l	42.99 ± 0.13 ij	56.83 ± 10.89 d–m	−27.65 ± 0.06 h–k	17.40 ± 0.02 e–l
TUTGMGH5	1.22 ± 0.05 h–l	0.35 ± 0.01 gh	1.57 ± 0.05 g–k	43.37 ± 0.06 e–g	52.92 ± 2.07 h–m	−28.11 ± 0.03 no	17.33 ± 0.11 e–l
TUTGMGH6	1.49 ± 0.07 c–j	0.85 ± 0.18 b	2.33 ± 0.19 b–d	43.81 ± 0.09 bc	65.14 ± 3.01 b–j	−27.74 ± 0.03 j–l	17.55 ± 0.27 d–l
TUTGMGH7	1.54 ± 0.01 b–i	0.50 ± 0.04–g	2.03 ± 0.03 c–h	43.72 ± 0.03 b–d	67.19 ± 0.35 c–g	−28.07 ± 0.01 no	15.57 ± 0.10 l
TUTGMGH8	1.27 ± 0.14 f–l	0.45 ± 0.02 d–h	1.59 ± 0.05 d–j	42.82 ± 0.10 jk	54.37 ± 6.13 g–m	−28.13 ± 0.06 o	16.54 ± 0.45 g–l
TUTGMGH9	1.61 ± 0.19 b–f	0.62 ± 0.01 b–e	2.23 ± 0.19 b–e	43.27 ± 0.09 f–i	69.64 ± 8.01 b–e	−27.19 ± 0.04 a	16.69 ± 0.10 f–l
TUTGMGH10	1.08 ± 0.02 l	0.39 ± 0.01 e–h	1.47 ± 0.01 l–n	42.76 ± 0.016 jk	46.04 ± 0.86 lm	−27.67 ± 0.03 h–l	16.17 ± 0.10 h–m
TUTGMGH11	1.53 ± 0.05 b–i	0.37 ± 0.02 f–h	1.90 ± 0.04 d–k	43.69 ± 0.11 b–d	66.85 ± 2.11 c–g	−27.25 ± 0.02 ab	17.07 ± 0.28 e–l
TUTGMGH12	1.38 ± 0.03 d–k	0.57 ± 0.02 c–g	1.95 ± 0.05 c–k	43.44 ± 0.02 d–g	59.81 ± 1.40 i–m	−27.76 ± 0.07 j–l	19.54 ± 0.24 cd
TUTGMGH13	1.21 ± 0.08 i–l	0.52 ± 0.002 c–g	1.73 ± 0.08 g–l	42.86 ± 0.011 j	51.86 ± 3.23 g–k	−27.69 ± 0.01 i–l	21.54 ± 0.07 b
TUTGMGH14	0.45 ± 0.05 mn	0.23 ± 0.01 h	0.68 ± 0.05 n	43.66 ± 0.11 b–e	19.44 ± 2.42 mn	−27.60 ± 0.05 g–j	19.86 ± 1.04 bc
TUTGMGH15	1.23 ± 0.09 g–l	0.61 ± 0.003 c–f	1.84 ± 0.09 e–l	43.45 ± 0.09 d–g	53.43 ± 3.96 h–l	−27.38 ± 0.09 b–f	19.87 ± 0.17 bc
TUTGMGH16	1.54 ± 0.03 b–i	0.61 ± 0.01 c–f	2.15 ± 0.04 c–g	43.01 ± 0.13 h–j	66.24 ± 1.60 b–i	−27.85 ± 0.01 lm	16.04 ± 0.09 i–l
TUTGMGH17	1.57 ± 0.01 b–h	0.67 ± 0.08 b–d	1.90 ± 0.40 c–f	43.56 ± 0.01 c–f	68.25 ± 0.28 b–g	−27.40 ± 0.02 b–f	18.78 ± 0.36 bcd
TUTGMGH18	0.72 ± 0.01 m	0.42 ± 0.05 d–h	1.14 ± 0.04 n	42.53 ± 0.35 kl	30.48 ± 0.38 no	−27.97 ± 0.05 m–o	18.67 ± 0.37 c–f
TUTGMGH19	1.56 ± 0.07 b–i	0.60 ± 0.01 c–f	2.16 ± 0.06 c–h	43.32 ± 0.07 fg	67.43 ± 2.83 b–h	−27.35 ± 0.02 a–e	17.68 ± 2.09 d–k
TUTGMGH20	1.62 ± 0.04 b–f	0.61 ± 0.03 c–f	2.23 ± 0.07 c–f	43.81 ± 0.03 bc	70.97 ± 1.74 a–d	−27.82 ± 0.03 k–m	15.68 ± 0.68 kl
TUTGMGH21	1.99 ± 0.10 a	0.72 ± 0.02 bc	2.70 ± 0.12 ab	42.77 ± 0.03 jk	84.98 ± 4.38 a	−27.37 ± 0.03 b–f	17.05 ± 0.10 e–l
TUTGMGH22	1.17 ± 0.09 j–l	0.64 ± 0.09 b–e	1.80 ± 0.15 f–l	41.88 ± 0.06 m	48.87 ± 3.73 j–l	−27.45 ± 0.05 c–g	17.18 ± 0.04 e–l
TUTGMGH23	1.55 ± 0.10 c–g	0.52 ± 0.07 c–g	2.07 ± 0.15 c–h	43.35 ± 0.15 e–g	70.75 ± 2.02 a–d	−28.44 ± 0.04 q	16.03 ± 1.29 i–l
TUTGMGH24	1.61 ± 0.02 b–f	0.51 ± 0.12 c–g	2.11 ± 0.11 c–i	44.42 ± 0.09 a	71.36 ± 0.84 a–d	−27.24 ± 0.01 ab	18.61 ± 0.36 e–g
TUTGMGH25	1.30 ± 0.03 e–k	0.61 ± 0.05 c–f	1.91 ± 0.02 d–k	42.43 ± 0.05 l	55.02 ± 1.38 e–l	−28.29 ± 0.04 p	15.91 ± 0.50 j–l
TUTGMGH26	0.60 ± 0.32 mn	0.60 ± 0.23 c–f	1.20 ± 0.44 mn	43.21 ± 0.12 g–i	26.00 ± 13.95 mn	−27.33 ± 0.02 a–c	16.87 ± 0.58 e–l
TUTGMGH27	1.48 ± 0.15 c–j	0.53 ± 0.02 c–g	2.00 ± 0.14 c–j	42.84 ± 0.04 j	63.25 ± 6.52 b–j	−27.34 ± 0.01 a–d	16.86 ± 0.58 e–l
TUTGMGH28	1.54 ± 0.15 b–i	0.58 ± 0.09 c–g	2.12 ± 0.20 c–h	43.90 ± 0.03 b	67.76 ± 6.50 b–h	−27.54 ± 0.01 f–i	17.55 ± 0.29 d–l
TUTGMGH29	1.48 ± 0.02 c–k	0.45 ± 0.01 d–h	1.93 ± 0.01 c–k	44.30 ± 0.02 a	65.56 ± 0.74 b–h	−27.74 ± 0.01 j–l	17.54 ± 0.67 d–l
TUTGMGH30	1.58 ± 0.09 b–f	0.72 ± 0.05 bc	2.30 ± 0.04 b–d	43.83 ± 0.01 bc	69.23 ± 3.79 b–f	−27.66 ± 0.10 h–k	17.84 ± 0.13 d–j
TUTGMGH31	1.13 ± 0.04 kl	0.41 ± 0.12–h	1.54 ± 0.07 k–m	42.84 ± 0.04 j	48.26 ± 1.83 k–m	−27.34 ± 0.11 a–d	16.86 ± 0.58 e–l
*Bradyrhizobium* strain WB74	1.07 ± 0.001 l	0.57 ± 0.04 c–g	1.64 ± 0004 i–m	43.88 ± 0.01 b	46.95 ± 0.02 l	−29.55 ± 0.06 r	21.59 ± 0.08 b
Uninoculated	0.33 ± 0.01 n	0.22 ± 0.07 h	0.54 ± 0.07 o	40.15 ± 0.02 n	13.12 ± 0.30 n	−27.77 ± 0.09 j–l	18.15 ± 0.98 c–i
5 mM KNO_3_	1.85 ± 0.07 ab	1.13 ± 0.08 a	2.97 ± 0.07 a	41.77 ± 0.01 m	77.14 ± 2.78 ab	−27.75 ± 0.01 j–l	23.59 ± 0.1.71 a
F-statistics	13.50 **	5.74 ***	15.74 **	68.00 **	13.18 **	70.00 **	8.90 ***

**Table 3:** Nodulation, relative symbiotic effectiveness, and N fixation of soybeans inoculated with different rhizobial isolates. Values (mean ± S.E.) followed by dissimilar letters are significant at ** *p* ≤ 0.01 or *** *p* ≤ 0.001, NA = not applicable.

Isolates	Nodule Number	Nodule Fresh Weight	Relative Symbiotic Effectiveness	N Concentration	N Content	δ^15^N	N-Fixed
	per Plant	g Plant^−1^	%	%	g Plant^−1^	‰	g Plant^−1^
TUTGMGH1	19 ± 0.88 h–j	0.64 ± 0.01 a	161 ± 8.63 ab	2.36 ± 0.01 f–i	4.07 ± 0.23 b–e	−2.02 ± 0.02 f–i	3.82 ± 0.23 b–e
TUTGMGH2	10 ± 0.58 k	0.52 ± 0.003 a–c	134 ± 2.55 b–i	2.41 ± 0.02 d–i	3.46 ± 0.06 c–h	−2.08 ± 0.05 f–k	3.21 ± 0.06 c–h
TUTGMGH3	37 ± 1.15 ab	0.44 ± 0.02 c–f	163 ± 8.63 ab	2.25 ± 0.02 g–i	3.91 ± 0.19 b–f	−1.76 ± 0.05 d	3.66 ± 0.19 b–f
TUTGMGH4	30 ± 1.15 b–e	0.61 ± 0.01 ab	124 ± 24.07 d–j	2.53 ± 0.03 b–h	3.36 ± 0.70 c–h	−2.15 ± 0.05 g–l	3.11 ± 0.70 c–h
TUTGMGH5	25 ± 6.35 e–i	0.34 ± 0.002 f–i	114 ± 4.32 f–j	2.52 ± 0.03 b–h	3.07 ± 0.14 f–h	−1.94 ± 0.02 d–g	2.82 ± 0.14 f–h
TUTGMGH6	18 ± 0.58 h–j	0.52 ± 0.001 bc	139 ± 6.14 b–h	2.60 ± 0.01 b–f	3.87 ± 0.18 b–f	−1.95 ± 0.02 d–g	3.62 ± 0.18 b–f
TUTGMGH7	20 ± 6.69 g–j	0.42 ± 0.02 c–g	144 ± 0.82 b–g	2.72 ± 0.03 a–c	4.18 ± 0.03 a–d	−2.28 ± 0.01 j–l	3.93 ± 0.03 a–d
TUTGMGH8	24 ± 1.73–j	0.44 ± 0.01 c–f	119 ± 1.49 e–j	2.61 ± 0.07 b–f	3.31 ± 0.40 d–h	−2.35 ± 0.01 l	3.06 ± 0.40 d–h
TUTGMGH9	35 ± 5.77 a–c	0.51 ± 0.10 b–d	150 ± 17.59 b–e	2.59 ± 0.02 b–f	4.16 ± 0.45 a–d	−2.17 ± 0.02 g–l	3.91 ± 0.45 a–d
TUTGMGH10	16 ± 0.58 jk	0.43 ± 0.01 c–f	101 ± 1.89 j	2.61 ± 0.04 b–f	2.81 ± 0.05 gh	−1.76 ± 0.02 d	2.56 ± 0.05 gh
TUTGMGH11	28 ± 0.88 c–g	0.43 ± 0.003 c–f	143 ± 4.32 b–g	2.59 ± 0.06 b–f	3.96 ± 0.15 b–f	−2.31 ± 0.03 kl	3.71 ± 0.15 b–f
TUTGMGH12	34 ± 0.33 bd	0.56 ± 0.01 ab	129 ± 2.97 c–j	2.27 ± 0.05 g–i	3.13 ± 0.11 e–h	−2.18 ± 0.01 g–l	2.88 ± 0.11 e–h
TUTGMGH13	30 ± 1.15 b–e	0.40 ± 0.03 d–h	133 ± 7.01 g–j	2.33 ± 0.08 f–i	2.79 ± 0.07 gh	−2.13 ± 0.05 g–l	2.54 ± 0.07 gh
TUTGMGH14	24 ± 2.03 e–j	0.36 ± 0.04 f–h	42 ± 5.13 k	2.23 ± 0.21 hi	0.99 ± 0.16 ij	−1.26 ± 0.03 c	0.75 ± 0.16 j
TUTGMGH15	26 ± 6.43 d–h	0.40 ± 0.03 d–h	115 ± 8.63 f–j	2.28 ± 0.03 g–i	2.81 ± 0.25 gh	−2.16 ± 0.02 g–l	2.56 ± 0.25 gh
TUTGMGH16	20 ± 0.58 g–j	0.43 ± 0.10 c–f	144 ± 3.24 b–g	2.70 ± 0.01 a–d	4.16 ± 0.10 a–d	−2.64 ± 0.28 m	3.91 ± 0.10 a–d
TUTGMGH17	25 ± 3.06 e–g	0.43 ± 0.01 c–f	146 ± 0.627 b–g	2.23 ± 0.02 g–i	3.50 ± 0.02 c–h	−2.11 ± 0.05 g–l	3.25 ± 0.02 c–h
TUTGMGH18	20 ± 3.76 g–j	0.19 ± 0.01 jk	67 ± 0.82 j	2.17 ± 0.11 i	1.56 ± 0.01 i	−2.14 ± 0.01 g–l	1.31 ± 0.10 i
TUTGMGH19	22 ± 0.58 e–j	0.55 ± 0.03 ab	145 ± 6.21 b–g	2.78 ± 0.06 ab	4.33 ± 0.24 a–c	−2.01 ± 0.03 e–h	4.08 ± 0.24 a–c
TUTGMGH20	43 ± 1.45 a	0.38 ± 0.01 e–h	151 ± 3.77 b–e	2.41 ± 0.11 d–i	3.90 ± 0.15 b–f	−2.29 ± 0.04 j–l	3.65 ± 0.15 b–f
TUTGMGH21	27 ± 0.33 c–h	0.50 ± 0.01 b–e	186 ± 9.51 a	2.53 ± 0.03 b–g	5.02 ± 0.23 a	−2.23 ± 0.03 h–l	4.77 ± 0.23 a
TUTGGH22	20 ± 0.88 g–j	0.39 ± 0.04 e–i	109 ± 8.24 h–j	2.34 ± 0.10 f–i	2.71 ± 0.14 gh	−2.23 ± 0.01 h–l	2.46 ± 0.14 gh
TUTGMGH23	23 ± 0.33 e–j	0.51 ± 0.01 b–e	153 ± 4.59 b–d	2.92 ± 0.04 a	4.77 ± 0.12 ab	−2.65 ± 0.24 m	4.53 ± 0.12 ab
TUTGMGH24	21 ± 0.33 f–j	0.38 ± 0.03 d–h	150 ± 1.89 b–e	2.39 ± 0.04 f–i	3.84 ± 0.10 b–f	−1.26 ± 0.03 c	3.59 ± 0.10 b–f
TUTGMGH25	35 ± 1.53 ab	0.58 ± 0.01 ab	121 ± 2.97 b–j	2.69 ± 0.07 a–e	3.49 ± 0.01 c–h	−2.79 ± 0.04 m	3.24 ± 0.01 c–h
TUTGMGH26	16 ± 0.88 i–k	0.18 ± 0.05 k	56 ± 29.93 k	2.46 ± 0.16 c–i	1.57 ± 0.92 i	−2.05 ± 0.001 f–j	1.32 ± 0.92 i
TUTGMGH27	16 ± 1.73 jk	0.24 ± 0.03 i–k	138 ± 14.30 b–i	2.37 ± 0.16 f–i	3.47 ± 0.35 c–h	−2.08 ± 0.03 f–k	3.22 ± 0.35 c–h
TUTGMGH28	29 ± 1.15 b–f	0.31 ± 0.01 g–i	144 ± 13.75 b–g	2.36 ± 0.15 f–i	3.64 ± 0.41 c–g	−1.86 ± 0.05 d–f	3.39 ± 0.41 c–g
TUTGMGH29	20 ± 0.33 g–j	0.43 ± 0.002 c–f	138 ± 1.62 b–i	2.43 ± 0.20 c–i	3.60 ± 0.29 c–g	−2.10 ± 0.05 f–k	3.35 ± 0.29 c–g
TUTGMGH30	21 ± 1.45 f–g	0.40 ± 0.08 d–h	147 ± 8.09 b–f	2.46 ± 0.02 c–i	3.88 ± 0.20 b–f	−1.79 ± 0.02 de	3.63 ± 0.20 b–f
TUTGMGH31	16 ± 0.58 jk	0.31 ± 0.01 g–i	105 ± 4.05 ij	2.37 ± 0.16 f–i	2.68 ± 0.29 gh	−2.08 ± 0.04 f–k	2.43 ± 0.29 gh
*Bradyrhizbium* strain WB74	30 ± 0.33 b–e	0.29 ± 0.01 h–j	100 ± 0.00 j	2.40 ± 0.16 e–i	2.57 ± 0.17 h	−2.26 ± 0.04 i–l	2.32 ± 0.17 h
Uninoculated	NA	NA	NA	1.13 ± 0.05 k	0.37 ± 0.02 j	+1.55 ± 0.01 b	NA
5 mM KNO_3_	NA	NA	NA	1.39 ± 0.02 j	2.57 ± 0.12 g	+2.16 ± 0.06 a	NA
F-statistics	8.05 ***	9.95 ***	9.90 ***	14.97 **	12.96 **	194.76 **	12.96 **

**Table 4:** Tolerance of introduced soybean rhizobial isolates to different levels of temperature, and NaCl and pH indicators. Scoring was completed as +++ = full growth, ++ = moderate growth, + = weak growth, and − no growth, as illustrated in Figure S1.

Isolates	Temperature °C						Salinity (NaCl) %						
	25	28	30	37	40	45	0.01	0.50	1	2	3	4	5
TUTGMGH1	++	+++	+++	++	++	++	+++	++	−	−	−	−	−
TUTGMGH2	++	+++	++	++	+++	++	+++	+	+	+	+	+	−
TUTGMGH3	+	+	+	+	+	+	+++	+++	+++	++	++	++	++
TUTGMGH4	+	+	++	+	+	+	+++	+++	++	+++	+++	−	−
TUTGMGH5	+++	+++	+++	++	−	−	+++	−	−	−	−	−	−
TUTGMGH6	+++	++	+++	+++	++	++	+++	+	+	+	+	+	+
TUTGMGH7	++	++	+++	+++	++	++	+++	+++	++	++	++	++	++
TUTGMGH8	+	++	++	+	+	+	+++	+++	+++	+++	+++	+++	+++
TUTGMGH9	++	+++	+++	++	−	−	+++	+++	+++	++	++	++	++
TUTGMGH10	++	+++	+++	+	+++	++	+++	+++	+++	+++	+++	−	−
TUTGMGH11	++	+++	++	+++	++	++	+++	−	−	−	−	−	−
TUTGMGH12	+++	++	++	+	+	+	+++	+++	+++	++	+	+	−
TUTGMGH13	+	++	++	+	+	++	+++	+++	+++	+++	+++	++	+
TUTGMGH14	++	++	+++	++	++	++	+++	++	+	+	+	+	−
TUTGMGH15	++	+++	+	+	+	++	+++	+++	++	++	++	++	+
TUTGMGH16	+++	+++	+++	+++	+++	+	+++	−	−	−	−	−	−
TUTGMGH17	+++	+++	+++	+++	+++	+++	+++	+	−	−	−	−	−
TUTGMGH18	+	++	+++	+++	+	+	+++	++	++	++	++	+	+
TUTGMGH19	++	+++	+++	++	++	++	+++	++	++	++	++	+	+
TUTGMGH20	+	++	+++	+++	++	+	+++	+++	++	++	++	+	+
TUTGMGH21	++	++	+++	+++	+++	++	+++	+++	++	++	++	+	+
TUTGMGH22	+++	+++	+++	++	+++	++	+++	+++	+++	+++	+++	++	++
TUTGMGH23	++	++	++	+	+	+	+++	+++	++	++	++	+	+
TUTGMGH24	+	++	++	+	++	++	+++	++	−	−	−	−	−
TUTGMGH25	+	+++	+++	+++	++	++	+++	+	−	−	−	−	−
TUTGMGH26	+	++	+++	+++	+++	++	+++	+++	++	++	++	++	−
TUTGMGH27	+	++	+++	+++	+++	++	+++	+++	+++	+++	++	++	++
TUTGMGH28	+	+++	+++	++	−	−	+++	+++	+++	+++	++	++	−
TUTGMGH29	+	+++	+	+	++	+	+++	++	++	+	+	+	+
TUTGMGH30	+	+++	++	+	+	+	+++	+++	++	++	+	+	+
TUTGMGH31	+	++	+++	++	+++	++	+++	+++	+++	+++	++	++	++

**Table 5:** Tolerance of isolates to drought and IAA-producing properties of rhizobial isolates nodulating soybeans. Values followed by dissimilar letters are significant at ** *p* ≤ 0.01. OD < 0.30 is highly sensitive to drought; OD = 0.30–0.39 is sensitive; OD = 0.40–0.50 is tolerant; and OD > 0.5 is highly tolerant.

Isolates	Drought				IAA
	Control	5%	15%	30%	(µg mL^−1^)
TUTGMGH1	0.170 ± 0.012 i–k	0.204 ± 0.003 f–h	0.084 ± 0.001 lm	0.078 ± 0.0003 e–h	8.59 ± 0.03 d
TUTGMGH2	0.333 ± 0.006 k–m	0.142 ± 0.001	0.088 ± 0.005 j–l	0.065 ± 0.006 gh	6.52 ± 0.15 f
TUTGMGH3	0.262 ± 0.001 h–j	0.258 ± 0.002 de	0.176 ± 0.004 b	0.069 ± 0.002 fgh	9.92 ± 0.10 b
TUTGMGH4	0.330 ± 0.036 d–g	0.210 ± 0.001 fg	0.077 ± 0.001 mn	0.075 ± 0.0003 e–h	8.59 ± 0.47 d
TUTGMGH5	0.238 ± 0.023 ij	0.103 ± 0.001 k–n	0.144 ± 0.003 e	0.083 ± 0.002 d–h	9.39 ± 0.16 bc
TUTGMGH6	0.634 ± 0.048 b	0.256 ± 0.005 de	0.114 ± 0.001 h	0.083 ± 0.002 d–h	8.74 ± 0.054 cd
TUTGMGH7	0.225 ± 0.003 i–k	0.131 ± 0.001 i–m	0.110 ± 0.002 h	0.084 ± 0.001 d–h	8.49 ± 0.15 de
TUTGMGH8	0.282 ± 0.0196 f–i	0.145 ± 0.005	0.135 ± 0.006 ef	0.086 ± 0.002 d–f	9.85 ± 0.01 b
TUTGMGH9	0.526 ± 0.015 c	0.523 ± 0.003 a	0.157 ± 0.009 d	0.067 ± 0.0003 f–h	8.30 ± 0.13 de
TUTGMGH10	0.277 ± 0.003 f–i	0.377 ± 0.067 c	0.171 ± 0.003 bc	0.154 ± 0.026 b	4.52 ± 0.03 g
TUTGMGH11	0.137 ± 0.002 mn	0.099 ± 0.001 l–n	0.085 ± 0.002 k–m	0.106 ± 00.004 c	8.65 ± 0.11 d
TUTGMGH12	0.639 ± 0.019 a	0.424 ± 0.002 b	0.243 ± 0.001 a	0.164 ± 0.001 a	8.11 ± 0.64 de
TUTGMGH13	0.338 ± 0.007 d–f	0.175 ± 0.0003 f–k	0.165 ± 0.002 cd	0.065 ± 0.002 h	7.75 ± 0.10 e
TUTGMGH14	0.105 ± 0.002 n	0.093 ± 0.0003 mn	0.057 ± 0.0003 p	0.089 ± 0.0003 c–e	4.01 ± 0.07 gh
TUTGMGH15	0.307 ± 0.003 e–h	0.082 ± 0.001 n	0.072 ± 0.001 no	0.085 ± 0.0003 d–g	7.75 ± 0.05 e
TUTGMGH16	0.447 ± 0.003 d	0.218 ± 0.0003 ef	0.164 ± 0.001 cd	0.098 ± 0.0003 cd	11.37 ± 0.23 a
TUTGMGH17	0.350 ± 0.002 d–f	0.172 ± 0.010 f–k	0.106 ± 0.0003 hi	0.041 ± 0.001 i	8.49 ± 0.04 de
TUTGMGH18	0.203 ± 0.042 j–l	0.096 ± 0.002 l–n	0.127 ± 0.001 fg	0.069 ± 0.001 f–h	4.16 ± 0.37 gh
TUTGMGH19	0.223 ± 0.007 i–k	0.461 ± 0.038 b	0.124 ± 0.003 g	0.142 ± 0.001 b	4.69 ± 0.35 g
TUTGMGH20	0.314 ± 0.005 e–h	0.128 ± 0.0003 i–n	0.097 ± 0.001 ij	0.075 ± 0.001 e–h	3.00 ± 0.14 ij
TUTGMGH21	0.270 ± 0.061 g–j	0.167 ± 0.006 g–j	0.166 ± 0.005 cd	0.101 ± 0.0123 cd	6.35 ± 0.03 f
TUTGMGH22	0.266 ± 0.002 g–j	0.086 ± 0.003 mn	0.05 ± 0.001 q	0.067 ± 0.0003 f–h	8.06 ± 0.69 de
TUTGMGH23	0.265 ± 0.013 h–j	0.176 ± 0.004 f–j	0.081 ± 0.001 lm	0.085 ± 0.00 d–1	4.48 ± 0.29 g
TUTGMGH24	0.156 ± 0.011 l–n	0.127 ± 0.006 j–n	0.090 ± 0.0003 j–l	0.091 ± 0.003 c–e	1.21 ± 0.14 k
TUTGMGH25	0.228 ± 0.012 i–k	0.128 ± 0.001 i–n	0.139 ± 0.003 e	0.078 ± 0.001 e–h	2.76 ± 0.04 jk
TUTGMGH26	0.258 ± 0.009 h–j	0.161 ± 0.005 h–j	0.096 ± 0.001 j	0.090 ± 0.001 c–e	0.98 ± 0.11 l
TUTGMGH27	0.235 ± 0.008 ij	0.159 ± 0.003 h–j	0.162 ± 0.001 cd	0.074 ± 0.002 e–h	3.02 ± 0.22 ij
TUTGMGH28	0.388 ± 0.002 de	0.159 ± 0.003 h–j	0.162 ± 0.001 cd	0.074 ± 0.002 e–h	2.14 ± 0.02 k
TUTGMGH29	0.269 ± 0.001 g–j	0.105 ± 0.0003 k–n	0.067 ± 0.001 o	0.044 ± 0.004 i	4.12 ± 0.07 gh
TUTGMGH30	0.227 ± 0.003 i–k	0.282 ± 0.003 d	0.094 ± 0.001 jk	0.089 ± 0.001 c–e	3.59 ± 0.26 hi
TUTGMGH31	0.257 ± 0.009 h–	0.160 ± 0.004 h–j	0.096 ± 0.001 j	0.091 ± 0.001 c–e	0.98 ± 0.11 l
F-statistics	41.294 **	62.881 **	207.34 **	22.857 **	135.02 **

**Table 6:** Growth response of soybean isolates to pH levels. Values followed by dissimilar letters are significant at ** *p* ≤ 0.01 and *** *p* ≤ 0.001, N/A = not applicable.

Isolates	4	5	6	7	8.5	pH Indicator (BTB)
TUTGHGM1	0.184 ± 0.002 k–m	0.466 ± 0.008 b	0.287 ± 0.079 de	0.178 ± 0.006 l	0.341 ± 0.055 d–h	Blue
TUTGHGM2	0.208 ± 0.008 h–m	0.151 ± 0.008 k	0.169 ± 0.004 m–o	0.250 ± 0.005 jk	0.355 ± 0.004 d–g	Yellow
TUTGHGM3	0.514 ± 0.019 b	0.452 ± 0.005 b	0.277 ± 0.009 d–f	0.245 ± 0.004 jk	0.284 ± 0.001 g–j	Yellow
TUTGHGM4	0.479 ± 0.062 b	0.349 ± 0.005 c	0.440 ± 0.009 b	0.429 ± 0.004 bc	0.475 ± 0.060 b	Blue
TUTGHGM5	0.158 ± 0.008 m–o	0.240 ± 0.002 fg	0.158 ± 0.002 no	0.237 ± 0.003 jk	0.258 ± 0.010 g–j	Blue
TUTGHGM6	0.230 ± 0.015 h–l	0.260 ± 0.026 ef	0.242 ± 0.011 e–j	0.238 ± 0.007 jk	0.417 ± 0.062 b–d	Yellow
TUTGHGM7	0.181 ± 0.002 lm	0.153 ± 0.002 k	0.242 ± 0.0002 e–j	0.222 ± 0.003 j–l	0.242 ± 0.009 h–j	Blue
TUTGHGM8	0.125 ± 0.005 no	0.279 ± 0.002 e	0.179 ± 0.004 l–o	0.416 ± 0.006 b–d	0.385 ± 0.063 b–f	Yellow
TUTGHGM9	0.585 ± 0.004 a	0.697 ± 0.008 a	0.556 ± 0.013 a	0.504 ± 0.003 a	0.388 ± 0.044 b–f	Yellow
TUTGHGM10	0.284 ± 0.045 fg	0.334 ± 0.005 cd	0.164 ± 0.007 no	0.421 ± 0.101 b–d	0.462 ± 0.0002 bc	Blue
TUTGHGM11	0.217 ± 0.004 h–l	0.199 ± 0.006 hi	0.231 ± 0.004 f–k	0.271 ± 0.005 h–j	0.220 ± 0.006 j	Blue
TUTGHGM12	0.423 ± 0.001 c	0.326 ± 0.006 cd	0.279 ± 0.002 d–f	0.383 ± 0.009 c–e	0.576 ± 0.005 a	Blue
TUTGHGM13	0.260 ± 0.001 gh	0.321 ± 0.005 d	0.204 ± 0.010 h–n	0.209 ± 0.007 kl	0.320 ± 0.004 d–j	Yellow
TUTGHGM14	0.256 ± 0.001 g–i	0.182 ± 0.015 j	0.183 ± 0.002 k–o	0.318 ± 0.010 f–h	0.301 ± 0.012 e–j	Blue
TUTGHGM15	0.328 ± 0.010 ef	0.233 ± 0.006 gh	0.256 ± 0.010 e–h	0.447 ± 0.08 b	0.240 ± 0.006 h–j	Yellow
TUTGHGM16	0.186 ± 0.010 j–m	0.156 ± 0.002 k	0.251 ± 0.003 e–i	0.341 ± 0.090 e–g	0.327 ± 0.019 d–i	Yellow
TUTGHGM17	0.330 ± 0.001 ef	0.062 ± 0.002 o	0.223 ± 0.003 j–l	0.335 ± 0.010 e–g	0.459 ± 0.060 bc	Blue
TUTGHGM18	0.128 ± 0.004 no	0.186 ± 0.001 ij	0.073 ± 0.0004 p	0.214 ± 0.002 j–l	0.397 ± 0.059 b–e	Yellow
TUTGHGM19	0.351 ± 0.016 de	0.329 ± 0.001 cd	0.376 ± 0.004 c	0.372 ± 0.007 d–f	0.311 ± 0.031 e–j	Blue
TUTGHGM20	0.341 ± 0.005 de	0.209 ± 0.004 hi	0.273 ± 0.002 e–g	0.229 ± 0.002 j–l	0.362 ± 0.010 c–f	Blue
TUTGHGM21	0.190 ± 0.020 j–m	0.126 ± 0.007 lm	0.170 ± 0.010 m–o	0.174 ± 0.007 l	0.402 ± 0.013 b–d	Blue
TUTGHGM22	0.240 ± 0.006 g–k	0.095 ± 0.002 n	0.144 ± 0.001 o	0.264 ± 0.003 i–k	0.268 ± 0.015 g–j	Blue
TUTGHGM23	0.318 ± 0.005 ef	0.225 ± 0.003 gh	0.199 ± 0.001 i–n	0.251 ± 0.005 jk	0.230 ± 0.004 ij	Yellow
TUTGHGM24	0.175 ± 0.012 l–n	0.134 ± 0.007 kl	0.195 ± 0.006 j–o	0.411 ± 0.001 b–d	0.380 ± 0.005 b–f	Blue
TUTGHGM25	0.192 ± 0.006 j–m	0.143 ± 0.015 kl	0.154 ± 0.005 no	0.329 ± 0.002 e–g	0.223 ± 0.006 ij	Blue
TUTGHGM26	0.213 ± 0.004 h–l	0.154 ± 0.008 k	0.206 ± 0.004 h–n	0.216 ± 0.007 j–l	0.320 ± 0.015 e–j	Yellow
TUTGHGM27	0.113 ± 0.002 o	0.109 ± 0.008 mn	0.218 ± 0.0004 h–m	0.225 ± 0.003 j–l	0.461 ± 0.014 bc	Blue
TUTGHGM28	0.384 ± 0.00001 cd	0.232 ± 0.010 gh	0.292 ± 0.002 de	0.224 ± 0.007 j–l	0.285 ± 0.018 f–j	Blue
TUTGHGM29	0.204 ± 0.003 i–m	0.153 ± 0.005 k	0.187 ± 0.008 k–o	0.225 ± 0.006 j–l	0.302 ± 0.032 e–j	Yellow
TUTGHGM30	0.240 ± 0.020 g–j	0.248 ± 0.007 fg	0.326 ± 0.005 d	0.311 ± 0.003 ghi	0.420 ± 0.005 b–d	Yellow
TUTGHGM31	0.123 ± 0.002 no	0.111 ± 0.008 mn	0.216 ± 0.0004 h–m	0.215 ± 0.003 j–l	0.461 ± 0.014 bc	Yellow
F-statistics	50.01 **	268.21 **	36.60 **	26.49 **	8.23 ***	N/A

## Data Availability

Data are contained within the article.

## Supplementary Materials

The following supporting information can be downloaded at: https://www.mdpi.com/article/10.3390/microorganisms13040876/s1. Table S1: the morphological characteristics of the 31 soybean nodulating rhizobial isolates; Table S2: correlation analysis between the shoot biomass of the soybeans and symbiotic N, as well as the gas-exchange parameters; Figure S1: colony morphology characteristics of the soybean rhizobia isolates on the yeast mannitol agar medium; Figure S2: a picture showing NaCl tolerance at different concentrations (A, 0.1 to 3% and B, 4 and 5%), exhibited by the test rhizobial isolates from Da. The numbers in each segment correspond to the number of the test rhizobial isolates, as preceded by the prefix TUTGMGH in Table 4.

## Abbreviations

The following abbreviations are used in this manuscript:

NaCl	Sodium Chloride
IAA	Indole acetic acid
PEG-6000	Polyethylene glycol-6000
BTB	Bromothymol blue
%RSE	Percent relative symbiotic effectiveness
O.D	Optical density
MDPI	Multidisciplinary Digital Publishing Institute
DOAJ	Directory of open access journals
