# Supplementary material for: Symbiotic N2 Fixation, Leaf Photosynthesis, and Abiotic Stress Tolerance of Native Rhizobia Isolated from Soybean Nodules at Da, Upper West Region, Ghana
Source: Microorganisms. 2025 Apr 11;13(4):876. doi: 10.3390/microorganisms13040876 (PMC12029937; doi:10.3390/microorganisms13040876)
Supplement: Supplementary file 1 [file microorganisms-13-00876-s001.zip › microorganisms-3540371-supplementary.pdf]

Table S1: Morphological characteristics of 31 soybean nodulating rhizobial isolates.

**Isolates**	**Growth days**	**Colour**	**Shape**	**Elevation**	**Opacity**	**Texture**	**Size (mm)**
TUTGMGH1	6	milky	Circular	Convex	Translucent	Gummy	1
TUTGMGH2	3	milky	Circular	Convex	Opaque	Watery	4
TUTGMGH3	4	White	Circular	Flat	Opaque	Gummy	1
TUTGMGH4	6	milky	Circular	Flat	Opaque	Gummy	2
TUTGMGH5	5	milky	Circular	Convex	Translucent	Watery	3
TUTGMGH6	5	White	Circular	Dome	Opaque	Dry	2
TUTGMGH7	4	milky	Circular	Flat	Translucent	Watery	3
TUTGMGH8	7	milky	Circular	Flat	Translucent	Gummy	1
TUTGMGH9	4	White	Circular	Convex	Opaque	Dry	3
TUTGMGH10	4	milky	Circular	Convex	Opaque	Gummy	1
TUTGMGH11	6	milky	Circular	Convex	Translucent	Watery	3
TUTGMGH12	4	White	Circular	Convex	Opaque	Dry	2
TUTGMGH13	7	milky	Irregular	Flat	Translucent	Gummy	2
TUTGMGH14	6	White	Circular	Flat	Opaque	Gummy	1
TUTGMGH15	5	white	Circular	Flat	Transparent	Dry	2
TUTGMGH16	8	milky	Circular	Convex	Translucent	Dry	2
TUTGMGH17	6	milky	Circular	Convex	Opaque	Gummy	2
TUTGMGH18	8	milky	Circular	Convex	Opaque	Gummy	2
TUTGMGH19	5	white	Circular	Convex	Opaque	Watery	3
TUTGMGH20	7	milky	Circular	Flat	Translucent	Gummy	1
TUTGMGH21	7	milky	Circular	Convex	Translucent	Watery	2
TUTGMGH22	5	milky	Circular	Convex	Translucent	Watery	4
TUTGMGH23	6	milky	Circular	Convex	Transparent	Dry	1
TUTGMGH24	6	milky	Irregular	Flat	Translucent	Gummy	1
TUTGMGH25	5	milky	Circular	Flat	Translucent	Gummy	1
TUTGMGH26	6	white	Circular	Convex	Opaque	Watery	4
TUTGMGH27	6	milky	Circular	Flat	Translucent	Gummy	2
TUTGMGH28	7	milky	Circular	Flat	Translucent	Gummy	2
TUTGMGH29	6	milky	Circular	Convex	Translucent	Gummy	3
TUTGMGH30	5	milky	Circular	Convex	Translucent	Watery	2
TUTGMGH31	6	white	Circular	Convex	Opaque	Watery	4

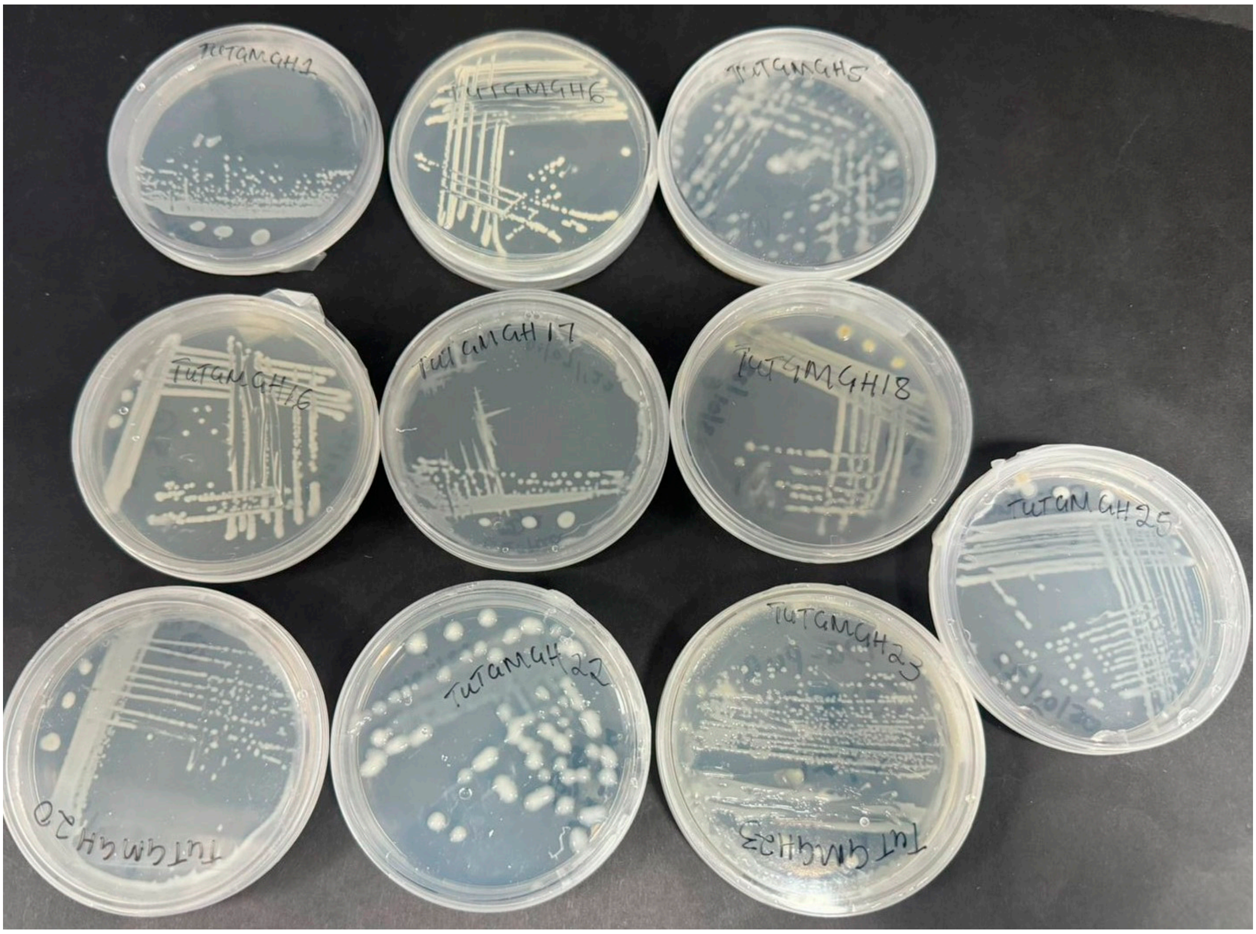

Figure S1: Colony morphology characteristics of soybean rhizobia isolates on yeast mannitol agar medium.

Table S2: Correlation analysis between shoot biomass of soybean and symbiotic N as well as gas-exchange parameters.

Parameters	r-value	p-values
Shoot dry matter x Nodule biomass	0.43	0.0001
Shoot dry matter x N concentration	0.31	0.001
Shoot dry matter x N fixed	0.91	0.001
$\delta^{13}C$ x C concentration	0.52	0.0001
$\delta^{13}C$ x gs	-0.28	0.005
$\delta^{13}C$ x E	-0.4	0.0001

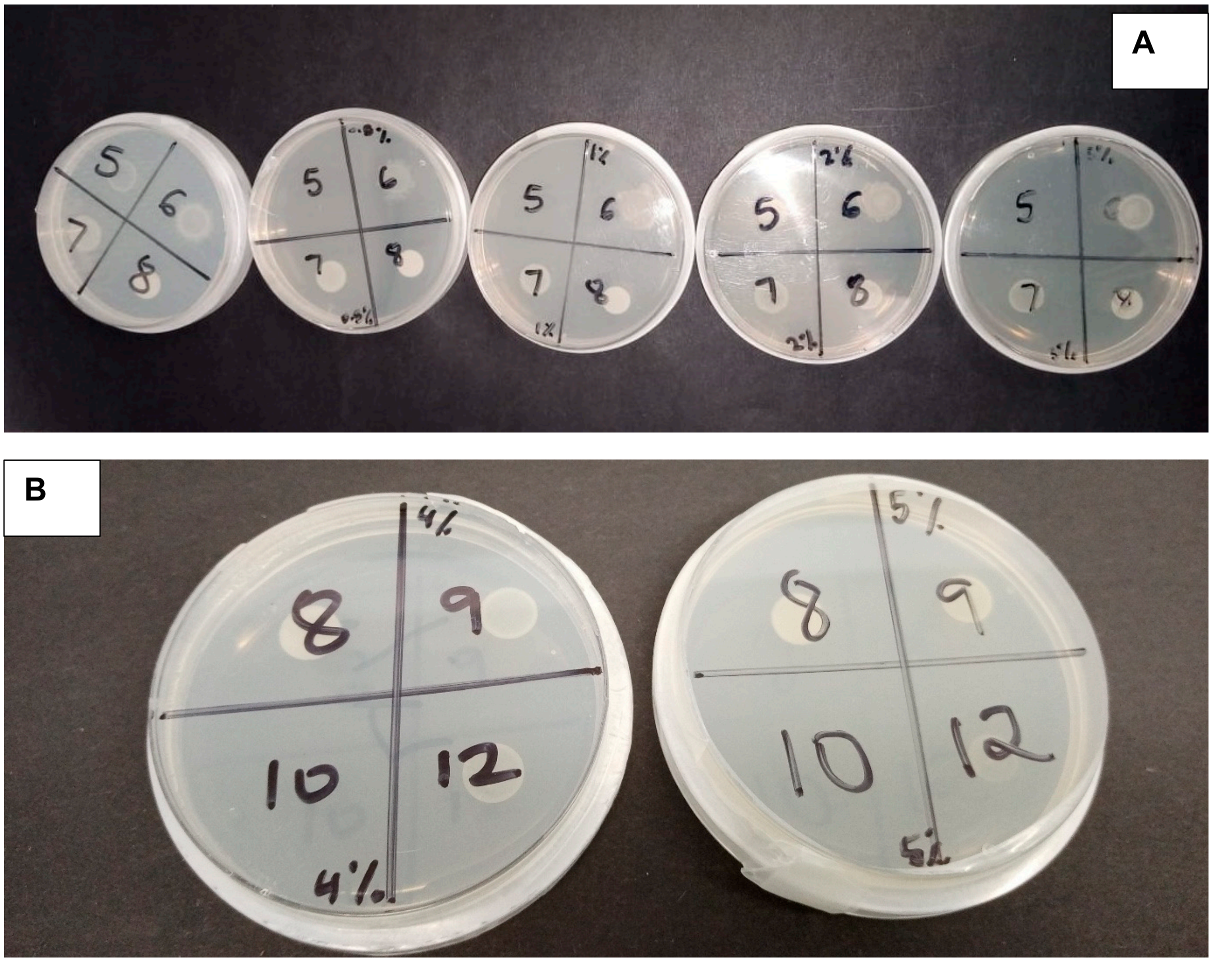

Figure S2: Picture showing NaCl tolerance at different concentrations (A, 0.1 to 3% and B, 4 and 5%) exhibited by the test rhizobial isolates from Da. The numbers in each segment correspond to the number of the test rhizobial isolates as preceded by the prefix TUTGMGH in Table 4.
